# Supplementary material for: Comparative efficacy and acceptability of interventions for major depression in older persons: protocol for Bayesian network meta-analysis
Source: BMJ Open. 2018 Jan 21;8(1):e019819. doi: 10.1136/bmjopen-2017-019819 (PMC5988061; doi:10.1136/bmjopen-2017-019819)
Supplement: Author's manuscript [file bmjopen-2017-019819.draft_revisions.pdf]

BMJ Open is committed to open peer review. As part of this commitment we make the peer review history of every article we publish publicly available.

When an article is published we post the peer reviewers' comments and the authors' responses online. We also post the versions of the paper that were used during peer review. These are the versions that the peer review comments apply to.

The versions of the paper that follow are the versions that were submitted during the peer review process. They are not the versions of record or the final published versions. They should not be cited or distributed as the published version of this manuscript.

BMJ Open is an open access journal and the full, final, typeset and author-corrected version of record of the manuscript is available on our site with no access controls, subscription charges or pay-per-view fees (http://bmjopen.bmj.com).

If you have any questions on BMJ Open's open peer review process please email editorial.bmjopen@bmj.com

BMJ Open

Comparative efficacy and acceptability of interventions for major depression in older persons: protocol for Bayesian network meta-analysis

Journal:	*BMJ Open*
Manuscript ID	bmjopen-2017-019819
Article Type:	Protocol
Date Submitted by the Author:	27-Sep-2017
Complete List of Authors:	Liew, Tau Ming; Institute of Mental Health, Department of Geriatric Psychiatry
**Primary Subject Heading**:	Mental health
Secondary Subject Heading:	Geriatric medicine
Keywords:	major depression, older person, efficacy, acceptability, network meta-analysis

SCHOLARONE™
Manuscripts

For peer review only

Comparative efficacy and acceptability of interventions for major depression in older persons: protocol for Bayesian network meta-analysis

Tau Ming Liew[1, 2]

[1]Department of Geriatric Psychiatry, Institute of Mental Health, Singapore

[2]Saw Swee Hock School of Public Health, National University of Singapore, Singapore

Correspondence to

Tau Ming Liew;

tau_ming_liew@imh.com.sg

Department of Geriatric Psychiatry, Institute of Mental Health,

10 Buangkok View, Singapore 539747.

For peer review only

Keywords: major depression; older person; efficacy; acceptability; network meta-analysis

Number of words (Abstract): 287

Number of words (main text): 2,009

Number of references: 26

Number of tables or figures: 1

For peer review only - http://bmjopen.bmj.com/site/about/guidelines.xhtml

ABSTRACT

Introduction: Major depression is a leading cause of disability, and has been associated with adverse effects in older persons. While many pharmacological and non-pharmacological interventions have been shown to be effective to address major depression in older persons, there has not been a meta-analysis that consolidates all the available interventions and compare the relative benefits of these available interventions. In this study, we aim to conduct a systematic review and network meta-analysis to compare the efficacy and acceptability of all the known pharmacological and non-pharmacological interventions for major depression in older persons.

Methods and analysis: We will search PubMed, Embase, PsycINFO, CINAHL, Web of Science, Scopus, Cochrane Central Register of Controlled Trials and references of other review articles for articles related to the keywords of 'randomized trial', 'major depression' and 'older persons'. Two reviewers will independently select the eligible articles. For each included article, the two reviewers will independently extract the data and assess the risk of bias using the Cochrane risk of bias tool. Bayesian network meta-analyses will be conducted to pool the efficacy (based on standardized mean difference of depression score) and all-cause attrition across all the included studies. The ranking probabilities for all interventions will be estimated and the hierarchy of each interventions will be summarized as surface under the cumulative ranking curve (SUCRA). Meta-regression and sub-group analyses will also be performed to evaluate the effect of study-level covariates. The quality of the evidence will be assessed using the Grading of Recommendations Assessment, Development and Evaluation (GRADE) approach.

Ethics and dissemination: The results will be disseminated through conference presentations and peer-reviewed publications. They will provide the consolidated evidence to

For peer review only

For peer review only - http://bmjopen.bmj.com/site/about/guidelines.xhtml

inform clinicians on the best choice of intervention to address major depression in older persons.

Trial registration number: International Prospective Register for Systematic Reviews (PROSPERO) temporary registration number 75756 (submitted on 30th August 2017).

For peer review only

For peer review only - http://bmjopen.bmj.com/site/about/guidelines.xhtml

STRENGTHS AND LIMITATIONS OF THIS STUDY

- This systematic review and meta-analysis will provide a comprehensive summary on the efficacy and acceptability of all available interventions for major depression in older persons.
- The results will provide the highest level of evidence to inform clinicians on the best choice of treatment, from among the many available pharmacological and non-pharmacological interventions.
- This protocol has been developed in accordance with the Preferred Reporting Items for Systematic Review and Meta-analysis Protocols (PRISMA-P) statement and has been submitted for registration with PROSPERO.
- The overall quality of evidence will be assessed using the Grading of Recommendations Assessment, Development and Evaluation (GRADE) approach.
- This systematic review will be limited to studies in English language.

For peer review only - http://bmjopen.bmj.com/site/about/guidelines.xhtml

INTRODUCTION

Rationale

Major depression has been identified by the World Health Organization as one of the leading cause of disability globally.[1 2] In older persons, its prevalence rates rise with the increase in medical comorbidities,[3] with reported rates of up to 5% in community-dwelling older persons,[3-5] 5 to 10% in primary care[3 6] and as high as 37% after critical care hospitalizations.[3 7] Major depression has a significant impact on the older populations and has been linked to higher risk of suicide,[4] myocardial infarction,[8] stroke,[9] all-cause mortality[4 10] and increasing health services utilization.[4]

A wide range of interventions have been available to treat major depression in older persons. These include pharmacological and non-pharmacological interventions such as antidepressants,[11] antipsychotics,[12] cognitive behavioural therapy,[13] problem solving therapy,[14] family interventions[15] and physical exercise.[16] Some of these interventions also have had recent meta-analyses confirming their efficacy when compared to control groups.[11 13 14 16] However, none of the meta-analyses had provided comparisons among all the pharmacological and non-pharmacological interventions to demonstrate the relative benefits of each intervention. It is unknown whether all the interventions have comparable efficacy and are equally suitable for older persons with major depression.

Objectives

For peer review only - http://bmjopen.bmj.com/site/about/guidelines.xhtml

In this study, we aim to conduct a systematic review and network meta-analysis to compare the efficacy and acceptability of all the available pharmacological and non-pharmacological interventions for major depression in older persons. The use of network meta-analysis allows us to pool the evidence on various interventions and rank their benefits relative to each other.[17] It also allows us to conduct indirect comparison of the different interventions, even when there is no direct evidence in the literature to allow head-to-head comparisons.

METHODS AND ANALYSIS

This protocol is developed in accordance with the Preferred Reporting Items for Systematic Review and Meta-analysis (PRISMA) statement.[18 19] It has also been submitted to the International Prospective Register of Systematic Reviews (PROSPERO) for registration (temporary registration number 75756, submitted on 30th August 2017).

Eligibility criteria

Participants and settings

We will include studies which recruit participants who are: (1) 60 years old and above; (2) diagnosed with major depression based on formal criteria by the Diagnostic and Statistical Manual of Mental Disorders (DSM) or International Classification of Diseases (ICD); and (3) having a current episode of major depression (that is, the participants are currently symptomatic and not in remission).

For peer review only - http://bmjopen.bmj.com/site/about/guidelines.xhtml

We will exclude studies which recruit participants with treatment-resistant depression, subthreshold depression, bipolar depression or psychotic depression. We will also exclude participants who have major depression but are currently asymptomatic or in remission.

Interventions

We will include studies which report on pharmacological interventions (such as antidepressants, antipsychotics or other class of medications) or non-pharmacological interventions (such as cognitive behavioural therapy, problem solving therapy, psychodynamic therapy or physical exercise). We will also include studies which report on combinations of any of these pharmacological and non-pharmacological interventions.

Comparators

We will accept control conditions such as placebo intervention, waiting-list, treatment as usual, as well as no intervention. We will also include studies with active comparators such as those which compare between two different interventions within the same studies.

Outcomes

We will only include a study if it reports at least one of the following outcome measures: (1) depression score at the immediate post-intervention period; (2) proportion of participants in each study arm with at least 50% improvement in depression score following intervention (response rate); (3) Clinical Global Impression–Improvement scale (CGI-I); or (4) all-cause attrition in each study arm at the immediate post-intervention period.

For peer review only - http://bmjopen.bmj.com/site/about/guidelines.xhtml

For peer review only

Study designs

We will only include randomized controlled trials (RCTs). The following study designs will be excluded: qualitative studies, observational studies, non-randomized trials, reviews, meta-analyses, case reports, case series, ecological studies, conference proceedings, letters, comments and policy papers.

Language and time frame

We will only include studies which are reported in the English language. Apart from that, we do not impose any time restriction to the publication year of the studies.

Information sources and search strategy

We will search PubMed, Embase, PsycINFO, CINAHL, Web of Science, Scopus and Cochrane Central Register of Controlled Trials for original articles related to the keywords of 'randomized trial', 'major depression' and 'older persons'. Our search strategy for PubMed is shown in Box 1. Similar search strategies will be used for the other databases. Additionally, we will also hand-search the references of review articles related to the topic to retrieve relevant articles which are not captured through our search of the electronic databases.

**Box 1. Search strategy for PubMed (MeSH, Medical Subject Headings)**
1. "Randomized Controlled Trial" [Publication Type] OR

For peer review only - http://bmjopen.bmj.com/site/about/guidelines.xhtml

2. "Randomized Controlled Trials as Topic"[Mesh] OR

3. "Random Allocation"[Mesh] OR

4. (random*[title/abstract] AND trial*[title/abstract]))

5. 1 or 2 or 3 or 4

6. "Depressive Disorder, Major/drug therapy"[Mesh] OR

7. "Depressive Disorder, Major/therapy"[Mesh] OR

8. (depress*[title] AND major[title]))

9. 6 or 7 or 8

10. elder*[title] OR

11. older[title] AND

12. (person*[title] OR people[title] OR adult*[title])) OR

13. 11 and 12

14. (late[title] AND life[title]) OR

15. geriatric[title])

16. 10 or 13 or 14 or 15

17. 5 and 9 and 16

Study selection

All potential articles will be retrieved and organized in a data management software (Endnote software, Thomson Reuters). After removing duplicate records, two reviewers will independently screen through the titles and abstracts to retain eligible articles. The first 10% of these titles and abstracts will be subjected to a calibration exercise between the two reviewers to ensure mutual agreement.

For peer review only - http://bmjopen.bmj.com/site/about/guidelines.xhtml

After completing the screening phase, articles that are deemed as relevant by at least one of the reviewers will be subjected to full-text review. The two reviewers will independently confirm the eligibility of these articles based on the full texts. The first 10% of these full texts will again undergo a calibration exercise by the two reviewers. After the full-text review, the included articles will be used for qualitative synthesis. The chance-corrected agreement between the two reviewers will be assessed using Cohen's Kappa (κ).

At any point during study selection, the reasons for excluding specific articles will be recorded. Moreover, any disagreements between the two reviewers will be resolved by discussion with a third reviewer.

Data extraction

Data from the selected studies will be extracted by two reviewers independently, and disagreements between the reviewers will be resolved by discussion with a third reviewer. The extracted data will include the following information:

1. Study identification (first author, year of publication, geographic location)
2. Study characteristics (study setting, study design, inclusion criteria, diagnostic criteria of major depression, sample size, study duration)
3. Participant characteristics (age, gender, education, number of comorbidities, Mini Mental State Examination score, baseline depression score, depression scale, duration of the current episode of major depression)
4. Characteristics of intervention and comparator (description, depression score, all-cause attrition)

For peer review only - http://bmjopen.bmj.com/site/about/guidelines.xhtml

The original authors of the RCTs will be contacted when the required data are not available in the published article.

Assessment of risk of bias

The risk of bias for each study will be assessed independently by two reviewers using the Cochrane risk of bias tool,[20] focusing on the key criteria of random sequence generation, allocation concealment, blinding of outcome assessment, completeness of outcome data and selective outcome reporting. Each criterion will be assigned a high, low or unclear risk of bias. Any disagreements between the two reviewers will be resolved by discussion with a third reviewer.

Outcome measures

Our primary outcomes are the efficacy and the acceptability of interventions. The efficacy will be based on difference in the depression scores between the intervention and comparator at the immediate post-intervention period, computed as standardized mean difference (SMD) for each RCT. The acceptability will be assessed by the relative risk (RR) of all-cause attrition at the immediate post-intervention period. This will be based on information extracted from each RCT, by subtracting those who were still available for data collection at the immediate post-intervention period from those who were randomized at the start of the RCT. Additionally, we will include a secondary outcome based on the RR of response rate at the immediate post-intervention period. We define response rate as the proportion of participants who have at least 50% improvement in depression score, or score much or very much improved on the Clinical Global Impression–Improvement scale (CGI-I<3).

For peer review only - http://bmjopen.bmj.com/site/about/guidelines.xhtml

For peer review only

Statistical analysis

We will conduct the network meta-analyses within a Bayesian framework using the Markov Chains Monte Carlo method. Bayesian analysis provides probabilistic distributions of our estimates-of-interest through large number of simulations, and hence produces results which have more intuitive interpretations. For example, Bayesian analysis generates the 95% credible interval which can be accurately interpreted as the range containing 95% of the estimates (based on the simulations). In the Bayesian analysis, we will run four Markov chains simultaneously with different arbitrarily chosen initial values and with non-informative priors. Each chain will have at least 10,000 simulations and at least the first 2,500 simulations will be discarded as burn-in. Convergence of the simulations will be assessed with the trace plots, kernel density plots and Gelman-Rubin-Brooks plots.

We will employ both fixed-effects and random-effects models in the Bayesian analyses, and will choose the final models based on the deviance information criterion (DIC). While there is no rule-of-thumb on what constitute significant improvements in DIC, we can take reference from the guideline commonly used in the analogous Akaike Information Criteria:[21] values which are lesser by at least 10 points indicate significantly better model-fit and parsimony. Hence, results from the random-effects model will be used if the random-effects model has DIC which is smaller by at least 10 points compared to the fixed-effect model. We will also compare the complexity of model between the fixed-effects and random-effects models using pD (an indicator which has higher value when a model is more complex), with preference for models which are more parsimonious (less complex). The global heterogeneity will be assessed with I^2 statistic. A common heterogeneity parameter will be

For peer review only - http://bmjopen.bmj.com/site/about/guidelines.xhtml

assumed in the random-effects model. Inconsistency between direct and indirect sources of evidence will be statistically assessed, by calculating the difference between direct and indirect estimates in each closed loop in the network.[22]

We will estimate the ranking probabilities for all interventions and show the results graphically in the form of rankograms and cumulative ranking probability plots. The hierarchy of interventions will be summarized as surface under the cumulative ranking curve (SUCRA) and presented in a scatterplot. SUCRAs have possible values ranging from 0% to 100%, with higher values indicating better efficacy or acceptability. Publication bias will be assessed with comparison-adjusted funnel plot.[23 24]

We will conduct meta-regression analyses to determine whether the results of our network meta-analyses will be affected by the following study-level covariates: sample size, study duration, inclusion criteria, study setting, study design and risk of bias. A covariate is considered as a significant moderator if the 95% credible interval of its beta coefficient in meta-regression does not include the value of zero. If a significant moderator is found, further subgroup analyses will then be conducted to assess the effect of this moderator.

The network meta-analyses will be conducted using JAGS (version 4.2.0), through the GeMTC package of R (version 3.3.1). The "Network Graphs" package in Stata statistical software (version 14.0) will also used to produce some of the figures in this study, such as the network plots, rankograms, cumulative ranking probability plots and comparison-adjusted funnel plots. [23 25]

Assessment of quality of evidence

For peer review only - http://bmjopen.bmj.com/site/about/guidelines.xhtml

We will use the Grading of Recommendations Assessment, Development and Evaluation (GRADE) approach to report the quality of evidence on efficacy and acceptability of interventions for major depression in older persons. Based on five key domains (methodology quality, directness of evidence, heterogeneity, precision of effect estimates and risk of publication bias), we will classify the quality of evidence in one of four levels – high, moderate, low and very low.[26]

ETHICS AND DISSEMINATION

This systematic review will provide the consolidated evidence to inform clinicians on the best choice of intervention, from among the many available options, to address major depression in older persons. This systematic review will be reported in accordance with the recommendations of PRISMA statement.[18 19] The results will be disseminated through conference presentations and publications in peer-reviewed journal.

FUNDING

TML was supported by a research fellowship under the Singapore Ministry of Health's National Medical Research Council (Grant number: NMRC/Fellowship/0030/2016). The funding source had no involvement in any part of the project.

For peer review only - http://bmjopen.bmj.com/site/about/guidelines.xhtml

COMPETING INTERESTS

None declared.

For peer review only

For peer review only - http://bmjopen.bmj.com/site/about/guidelines.xhtml

REFERENCES

1. World Health Organization. The global burden of disease: 2004 update. Switzerland: World Health Organization.

2. World Health Organization. Global Health Estimates 2015: Disease burden by Cause, Age, Sex, by Country and by Region, 2000-2015. Geneva, 2016.

3. Taylor WD. Clinical practice. Depression in the elderly. *The New England journal of medicine* 2014;371(13):1228-36. doi: 10.1056/NEJMcp1402180 [published Online First: 2014/09/25]

4. Blazer DG. Depression in late life: review and commentary. *The journals of gerontology Series A, Biological sciences and medical sciences* 2003;58(3):249-65. [published Online First: 2003/03/14]

5. Volkert J, Schulz H, Härter M, et al. The prevalence of mental disorders in older people in Western countries – a meta-analysis. *Ageing Research Reviews* 2013;12(1):339-53. doi: http://dx.doi.org/10.1016/j.arr.2012.09.004

6. Lyness JM, Caine ED, King DA, et al. Psychiatric Disorders in Older Primary Care Patients. *Journal of general internal medicine* 1999;14(4):249-54. doi: 10.1046/j.1525-1497.1999.00326.x

7. Jackson JC, Pandharipande PP, Girard TD, et al. Depression, Posttraumatic Stress Disorder, and Functional Disability in Survivors of Critical Illness: results from the BRAIN ICU (Bringing to light the Risk Factors And Incidence of Neuropsychological dysfunction in ICU survivors) Investigation: A Longitudinal Cohort Study. *The lancet Respiratory medicine* 2014;2(5):369-79. doi: 10.1016/S2213-2600(14)70051-7

For peer review only - http://bmjopen.bmj.com/site/about/guidelines.xhtml

8. Gan Y, Gong Y, Tong X, et al. Depression and the risk of coronary heart disease: a meta-analysis of prospective cohort studies. *BMC psychiatry* 2014;14:371. doi: 10.1186/s12888-014-0371-z [published Online First: 2014/12/30]

9. Pan A, Sun Q, Okereke OI, et al. Depression and risk of stroke morbidity and mortality: a meta-analysis and systematic review. *Jama* 2011;306(11):1241-9. doi: 10.1001/jama.2011.1282 [published Online First: 2011/09/22]

10. Cuijpers P, Vogelzangs N, Twisk J, et al. Comprehensive meta-analysis of excess mortality in depression in the general community versus patients with specific illnesses. *The American journal of psychiatry* 2014;171(4):453-62. doi: 10.1176/appi.ajp.2013.13030325 [published Online First: 2014/01/18]

11. Tham A, Jonsson U, Andersson G, et al. Efficacy and tolerability of antidepressants in people aged 65 years or older with major depressive disorder – A systematic review and a meta-analysis. *Journal of affective disorders* 2016;205:1-12. doi: http://dx.doi.org/10.1016/j.jad.2016.06.013

12. Katila H, Mezhebovsky I, Mulroy A, et al. Randomized, double-blind study of the efficacy and tolerability of extended release quetiapine fumarate (quetiapine XR) monotherapy in elderly patients with major depressive disorder. *The American journal of geriatric psychiatry : official journal of the American Association for Geriatric Psychiatry* 2013;21(8):769-84. doi: 10.1016/j.jagp.2013.01.010 [published Online First: 2013/04/10]

13. Gould RL, Coulson MC, Howard RJ. Cognitive behavioral therapy for depression in older people: a meta-analysis and meta-regression of randomized controlled trials. *Journal of the American Geriatrics Society* 2012;60(10):1817-30. doi: 10.1111/j.1532-5415.2012.04166.x [published Online First: 2012/09/26]

For peer review only - http://bmjopen.bmj.com/site/about/guidelines.xhtml

14. Kirkham JG, Choi N, Seitz DP. Meta-analysis of problem solving therapy for the treatment of major depressive disorder in older adults. *International journal of geriatric psychiatry* 2016;31(5):526-35. doi: 10.1002/gps.4358 [published Online First: 2015/10/06]

15. Stahl ST, Rodakowski J, Saghafi EM, et al. Systematic review of dyadic and family oriented interventions for late life depression. *International journal of geriatric psychiatry* 2016;31(9):963-73. doi: 10.1002/gps.4434

16. Schuch FB, Vancampfort D, Rosenbaum S, et al. Exercise for depression in older adults: a meta-analysis of randomized controlled trials adjusting for publication bias. *Revista brasileira de psiquiatria (Sao Paulo, Brazil : 1999)* 2016;38(3):247-54. doi: 10.1590/1516-4446-2016-1915 [published Online First: 2016/09/10]

17. Tonin FS, Rotta I, Mendes AM, et al. Network meta-analysis: a technique to gather evidence from direct and indirect comparisons. *Pharmacy practice* 2017;15(1):943. doi: 10.18549/PharmPract.2017.01.943 [published Online First: 2017/05/16]

18. Moher D, Shamseer L, Clarke M, et al. Preferred reporting items for systematic review and meta-analysis protocols (PRISMA-P) 2015 statement. *Systematic reviews* 2015;4:1. doi: 10.1186/2046-4053-4-1 [published Online First: 2015/01/03]

19. Shamseer L, Moher D, Clarke M, et al. Preferred reporting items for systematic review and meta-analysis protocols (PRISMA-P) 2015: elaboration and explanation. *BMJ (Clinical research ed)* 2015;349:g7647. doi: 10.1136/bmj.g7647 [published Online First: 2015/01/04]

20. Higgins JPT, Green S. Cochrane Handbook for Systematic Reviews of Interventions: Wiley 2011.

21. Burnham KP, Anderson DR. Model Selection and Multimodel Inference: A Practical Information-Theoretic Approach: Springer New York 2003.

For peer review only - http://bmjopen.bmj.com/site/about/guidelines.xhtml

22. Dias S, Welton NJ, Caldwell DM, et al. Checking consistency in mixed treatment comparison meta-analysis. *Statistics in medicine* 2010;29(7-8):932-44. doi: 10.1002/sim.3767 [published Online First: 2010/03/10]

23. Chaimani A, Higgins JP, Mavridis D, et al. Graphical tools for network meta-analysis in STATA. *PloS one* 2013;8(10):e76654. doi: 10.1371/journal.pone.0076654 [published Online First: 2013/10/08]

24. Peters JL, Sutton AJ, Jones DR, et al. Assessing publication bias in meta-analyses in the presence of between-study heterogeneity. *Journal of the Royal Statistical Society: Series A (Statistics in Society)* 2010;173(3):575-91. doi: 10.1111/j.1467-985X.2009.00629.x

25. Chaimani A, Salanti G. Visualizing assumptions and results in network meta-analysis: The network graphs package. *Stata Journal* 2015;15(4):905-50.

26. Schunemann H, Brozek J, Guyatt G, et al. GRADE handbook for grading quality of evidence and strength of recommendation 2013. Available from: http://gdt.guidelinedevelopment.org/app/ (assessed 22 Nov 2016).

For peer review only

For peer review only - http://bmjopen.bmj.com/site/about/guidelines.xhtml

PRISMA-P (Preferred Reporting Items for Systematic review and Meta-Analysis Protocols) 2015 checklist: recommended items to address in a systematic review protocol*

Section and topic	Item No	Checklist item	Page (Line)
**ADMINISTRATIVE INFORMATION**			
Title:			
Identification	1a	Identify the report as a protocol of a systematic review	1 (3)
Update	1b	If the protocol is for an update of a previous systematic review, identify as such	Not applicable
Registration	2	If registered, provide the name of the registry (such as PROSPERO) and registration number	3 (7)
Authors:			
Contact	3a	Provide name, institutional affiliation, e-mail address of all protocol authors; provide physical mailing address of corresponding author	1 (21)
Contributions	3b	Describe contributions of protocol authors and identify the guarantor of the review	Not applicable
Amendments	4	If the protocol represents an amendment of a previously completed or published protocol, identify as such and list changes; otherwise, state plan for documenting important protocol amendments	Not applicable
Support:			
Sources	5a	Indicate sources of financial or other support for the review	14 (41)
Sponsor	5b	Provide name for the review funder and/or sponsor	Not applicable
Role of sponsor or funder	5c	Describe roles of funder(s), sponsor(s), and/or institution(s), if any, in developing the protocol	14 (47)
**INTRODUCTION**			
Rationale	6	Describe the rationale for the review in the context of what is already known	5 (8)
Objectives	7	Provide an explicit statement of the question(s) the review will address with reference to participants, interventions, comparators, and outcomes (PICO)	5 (53)
**METHODS**			
Eligibility criteria	8	Specify the study characteristics (such as PICO, study design, setting, time frame) and report characteristics (such as years considered, language, publication status) to be used as criteria for eligibility for the review	6 (36)
Information sources	9	Describe all intended information sources (such as electronic databases, contact with study authors, trial registers or other grey literature sources) with planned dates of coverage	8 (32)
Search strategy	10	Present draft of search strategy to be used for at least one electronic database, including planned limits, such that it could be repeated	8 (54)

For peer review only

Study records:			
Data management	11a	Describe the mechanism(s) that will be used to manage records and data throughout the review	9 (45)
Selection process	11b	State the process that will be used for selecting studies (such as two independent reviewers) through each phase of the review (that is, screening, eligibility and inclusion in meta-analysis)	10 (3)
Data collection process	11c	Describe planned method of extracting data from reports (such as piloting forms, done independently, in duplicate), any processes for obtaining and confirming data from investigators	10 (32)
Data items	12	List and define all variables for which data will be sought (such as PICO items, funding sources), any pre-planned data assumptions and simplifications	10 (37)
Outcomes and prioritization	13	List and define all outcomes for which data will be sought, including prioritization of main and additional outcomes, with rationale	11 (30)
Risk of bias in individual studies	14	Describe anticipated methods for assessing risk of bias of individual studies, including whether this will be done at the outcome or study level, or both; state how this information will be used in data synthesis	11 (10)
Data synthesis	15a	Describe criteria under which study data will be quantitatively synthesised	Not applicable
	15b	If data are appropriate for quantitative synthesis, describe planned summary measures, methods of handling data and methods of combining data from studies, including any planned exploration of consistency (such as I^2, Kendall's τ)	12 (5)
	15c	Describe any proposed additional analyses (such as sensitivity or subgroup analyses, meta-regression)	13 (27)
	15d	If quantitative synthesis is not appropriate, describe the type of summary planned	Not applicable
Meta-bias(es)	16	Specify any planned assessment of meta-bias(es) (such as publication bias across studies, selective reporting within studies)	13 (22)
Confidence in cumulative evidence	17	Describe how the strength of the body of evidence will be assessed (such as GRADE)	14 (3)

*** It is strongly recommended that this checklist be read in conjunction with the PRISMA-P Explanation and Elaboration (cite when available) for important clarification on the items. Amendments to a review protocol should be tracked and dated. The copyright for PRISMA-P (including checklist) is held by the PRISMA-P Group and is distributed under a Creative Commons Attribution Licence 4.0.**

From: Shamseer L, Moher D, Clarke M, Ghersi D, Liberati A, Petticrew M, Shekelle P, Stewart L, PRISMA-P Group. Preferred reporting items for systematic review and meta-analysis protocols (PRISMA-P) 2015: elaboration and explanation. BMJ. 2015 Jan 2;349(jan02 1):g7647.

For peer review only

BMJ Open

Comparative efficacy and acceptability of interventions for major depression in older persons: protocol for Bayesian network meta-analysis

Journal:	*BMJ Open*
Manuscript ID	bmjopen-2017-019819.R1
Article Type:	Protocol
Date Submitted by the Author:	28-Oct-2017
Complete List of Authors:	Liew, Tau Ming; Institute of Mental Health, Department of Geriatric Psychiatry<br>Lee, Cia Sin; SingHealth Polyclinics, Sengkang Polyclinic
**Primary Subject Heading**:	Mental health
Secondary Subject Heading:	Geriatric medicine
Keywords:	major depression, older person, efficacy, acceptability, network meta-analysis

SCHOLARONE™
Manuscripts

Comparative efficacy and acceptability of interventions for major depression in older persons: protocol for Bayesian network meta-analysis

Tau Ming Liew[1, 2], Cia Sin Lee[3]

[1]Department of Geriatric Psychiatry, Institute of Mental Health, Singapore

[2]Saw Swee Hock School of Public Health, National University of Singapore

[3]SingHealth Polyclinics, Singapore

Correspondence to

Tau Ming Liew;

tau_ming_liew@imh.com.sg

Department of Geriatric Psychiatry, Institute of Mental Health,

10 Buangkok View, Singapore 539747.

Keywords: major depression; older person; efficacy; acceptability; network meta-analysis

Number of words (Abstract): 290

Number of words (main text): 2,946

Number of references: 36

Number of tables or figures: 1

For peer review only - http://bmjopen.bmj.com/site/about/guidelines.xhtml

For peer review only

ABSTRACT

Introduction: Major depression is a leading cause of disability, and has been associated with adverse effects in older persons. While many pharmacological and non-pharmacological interventions have been shown to be effective to address major depression in older persons, there has not been a meta-analysis that consolidates all the available interventions and compare the relative benefits of these available interventions. In this study, we aim to conduct a systematic review and network meta-analysis to compare the efficacy and acceptability of all the known pharmacological and non-pharmacological interventions for major depression in older persons.

Methods and analysis: We will search MEDLINE, Embase, PsycINFO, Cumulative Index to Nursing and Allied Health, Cochrane Central Register of Controlled Trials and references of other review articles for articles related to the keywords of 'randomized trial', 'major depression', 'older persons' and 'treatments'. Two reviewers will independently select the eligible articles. For each included article, the two reviewers will independently extract the data and assess the risk of bias using the Cochrane revised tool for Risk of Bias. Bayesian network meta-analyses will be conducted to pool the depression scores (based on standardized mean difference) and the all-cause discontinuation across all included studies. The ranking probabilities for all interventions will be estimated and the hierarchy of each interventions will be summarized as surface under the cumulative ranking curve (SUCRA). Meta-regression and sub-group analyses will also be performed to evaluate the effect of study-level covariates. The quality of the evidence will be assessed using the Grading of Recommendations Assessment, Development and Evaluation (GRADE) approach.

Ethics and dissemination: The results will be disseminated through conference presentations and peer-reviewed publications. They will provide the consolidated evidence to

For peer review only - http://bmjopen.bmj.com/site/about/guidelines.xhtml

inform clinicians on the best choice of intervention to address major depression in older persons.

Trial registration number: International Prospective Register for Systematic Reviews (PROSPERO) number CRD42017075756.

For peer review only

For peer review only - http://bmjopen.bmj.com/site/about/guidelines.xhtml

STRENGTHS AND LIMITATIONS OF THIS STUDY

- This systematic review and meta-analysis will provide a comprehensive summary on the efficacy and acceptability of all available interventions for major depression in older persons.
- The results will provide the highest level of evidence to inform clinicians on the best choice of treatment, from among the many available pharmacological and non-pharmacological interventions.
- This protocol has been developed in accordance with the Preferred Reporting Items for Systematic Review and Meta-analysis Protocols (PRISMA-P) statement and has been registered with PROSPERO.
- The overall quality of evidence will be assessed using the Grading of Recommendations Assessment, Development and Evaluation (GRADE) approach.
- This systematic review will be limited to studies which are reported in English language and have been peer-reviewed.

For peer review only

INTRODUCTION

Rationale

Major depression has been identified by the World Health Organization as one of the leading cause of disability globally.[1 2] In older persons, its prevalence rates rise with the increase in medical comorbidities,[3] with reported rates of up to 5% in community-dwelling older persons,[3-5] 5 to 10% in primary care[3 6] and as high as 37% after critical care hospitalizations.[3 7] Major depression has a significant impact on the older populations and has been linked to higher risk of suicide,[4] myocardial infarction,[8] stroke,[9] all-cause mortality[4 10] and increasing health services utilization.[4]

Many of the interventions for major depression in older persons have had recent meta-analyses confirming their efficacy when compared to control groups. These include antidepressants,[11-14] cognitive behavioural therapy,[15] problem solving therapy,[16] psychological interventions in general,[17-19] and the various forms of non-pharmacological interventions.[20-22] However, none of the meta-analyses had compared all the pharmacological and non-pharmacological interventions together to demonstrate the relative benefits of each intervention. It is unknown whether the different types of pharmacological and non-pharmacological interventions have comparable efficacy and are equally suitable for older persons with major depression.

Objectives

For peer review only - http://bmjopen.bmj.com/site/about/guidelines.xhtml

In this study, we aim to conduct a systematic review and network meta-analysis to compare the efficacy and acceptability of all the available pharmacological and non-pharmacological interventions for major depression in older persons. The use of network meta-analysis allows us to pool the evidence on various interventions and rank their benefits relative to each other.[23] It also allows us to conduct indirect comparison of the different interventions, even when there is no direct evidence in the literature to allow head-to-head comparisons.

METHODS AND ANALYSIS

This protocol is developed in accordance with the Preferred Reporting Items for Systematic Review and Meta-analysis (PRISMA) statement.[24 25] It has also been registered with the International Prospective Register of Systematic Reviews (PROSPERO) (registration number CRD42017075756).

Eligibility criteria

Participants and settings

We will include studies which recruited participants who were:

- 60 years old and above;
- diagnosed with major depression based on formal criteria by the Diagnostic and Statistical Manual of Mental Disorders (DSM) or International Classification of Diseases (ICD); and

For peer review only - http://bmjopen.bmj.com/site/about/guidelines.xhtml
For peer review only

- having a current episode of major depression (that is, the participants were symptomatic and not in remission at the point of recruitment; and the intervention was not intended primarily for the prevention of future relapses).

We will exclude studies which recruited participants with treatment-resistant depression, subthreshold depression, bipolar depression, depression in dementia or psychotic depression. We will not include maintenance studies for major depression as such studies primarily focused on the prevention of relapses in participants who had been asymptomatic or in remission at the point of recruitment.

Interventions

We will include studies with pharmacological interventions, including but not limited to:

- Antidepressants such as citalopram, sertraline, venlafaxine or mirtazapine;
- Antipsychotics such as risperidone, quetiapine, olanzapine or aripiprazole;
- Mood-stabilizers such as valproate, carbamazepine, lithium or gabapentin.

We will include studies with non-pharmacological interventions, including but not limited to:

- Psychological interventions such as cognitive behavioural therapy, interpersonal therapy, problem solving therapy, psychodynamic therapy or family interventions;
- Procedural interventions such as electroconvulsive therapy, transcranial magnetic stimulation, transcranial direct-current stimulation or bright light therapy.

We will also include studies which reported on combinations of any of these pharmacological and non-pharmacological interventions.

For peer review only - http://bmjopen.bmj.com/site/about/guidelines.xhtml

We will exclude studies which focused primarily on health service models of care but were not related to any modality of intervention, such as studies which evaluated the effectiveness of home treatment, training of general practitioners, multidisciplinary approach or stepped-care approach.

Comparators

We will accept control conditions such as placebo intervention, waiting-list, treatment as usual, as well as no intervention. We will also include studies with active comparators such as those which compare between two different interventions within the same studies.

Outcomes

We will only include a study if it reports the depression scores or the all-cause discontinuation in each study arm following intervention.

Study designs and publication types

We will only include randomized controlled trials (RCTs) which aimed to demonstrate the superiority of a treatment to another (also known as superiority trials), and will not include equivalence or non-inferiority trials. The following study designs or publication types will also be excluded: qualitative studies, observational studies, meta-analyses, case reports, case series, ecological studies and policy papers. We intend to include only higher-quality

For peer review only - http://bmjopen.bmj.com/site/about/guidelines.xhtml

evidence and hence will exclude non-randomized trials and publications which were not peer-reviewed (such as conference proceedings, letters and comments).

Language and time frame

We will only include studies which are reported in the English language. Apart from that, we do not impose any time restriction to the publication year of the studies. The search of databases will be conducted in January 2018.

Information sources and search strategy

We will search MEDLINE, Embase, PsycINFO, Cumulative Index to Nursing and Allied Health (CINAHL) and Cochrane Central Register of Controlled Trials (CENTRAL) for original articles related to the keywords of 'randomized trial', 'major depression', 'older persons' and 'treatments'. Our search strategy for MEDLINE is shown in Box 1. Similar search strategies will be used for the other databases. Additionally, we will also hand-search the references of review articles related to the topic to retrieve relevant articles which are not captured through our search of the electronic databases. We will examine the full text of the relevant articles and include the respective articles if they meet our eligibility criteria.

Box 1. Search strategy for MEDLINE (via Ovid interface)

1. *Therapeutics/ OR *Drug Therapy/ OR *Psychotropic Drugs/ OR *Antidepressive Agents/ OR *Antipsychotic Agents/ OR *Antimanic Agents/ OR *Anticonvulsants/ OR *Psychotherapy/ OR *Electroconvulsive Therapy/ OR *Transcranial Magnetic Stimulation/ OR *Transcranial Direct Current Stimulation/ OR *Phototherapy/
2. (antidepressant* OR selective serotonin reuptake inhibitor OR SSRI OR citalopram OR fluoxetine OR paroxetine OR sertraline OR escitalopram OR fluvoxamine OR (serotonin adj2 epinephrine adj reuptake adj inhibitor) OR SNRI OR venlafaxine OR desvenlafaxine OR duloxetine OR milnacipran OR reboxetine OR bupropion OR noradrenergic and specific serotonergic antidepressant OR NaSSA OR mirtazapine OR TCA OR tricyclic OR amersergide

For peer review only - http://bmjopen.bmj.com/site/about/guidelines.xhtml

OR amineptine OR amitriptyline OR amoxapine OR butriptyline OR chlorpoxiten OR clomipramine OR clorimipramine OR demexiptiline OR desipramine OR dibenzipin OR dothiepin OR doxepin OR imipramine OR lofepramine OR melitracen OR metapramine OR nortriptyline OR noxiptiline OR opipramol OR protriptyline OR quinupramine OR trimipramine OR tianeptine OR trazodone OR nefazodone OR agomelatine).ab,ti

3. (antipsychotic* OR haloperidol OR trifluoperazine OR benperidol OR chlorprothixene OR flupenthixol OR clopenthixol OR chlorpromazine OR prochlorperazine OR sulpiride OR periciazine OR perphenazine OR pimozide OR promazine OR fluspirilene OR methotrimeprazine OR risperidone OR paliperidone OR quetiapine OR olanzapine OR amisulpride OR amisulpiride OR aripiprazole OR clozapine OR sertindole OR zotepine).ab,ti
4. ((mood adj stabili*) OR (antimanic adj (agent* OR drug*)) OR anticonvuls* OR anti convuls* OR carbamazepine OR ethosuximide OR gabapentin OR lacosamide OR lamotrigine OR levetiracetam OR lithium OR oxcarbazepine OR phenobarbital OR phenytoin OR pregabalin OR rufinamide OR tiagabine OR topiramate OR valproic acid OR valproate OR verapamil OR vigabatrin OR zonisamide).ab,ti
5. (psychotherap* OR therap* OR cognitive behavio* therapy OR cognitive therapy OR behavio* therapy OR interpersonal therapy OR inter-personal therapy OR problem solving therapy OR problem-solving therapy OR (family adj (therapy OR intervention)) OR bibliotherapy OR mindful* OR (group adj (therapy OR intervention)) OR psychodynamic OR psychoanalytic OR emotion-focused OR emotion focused OR reminiscen* OR life review OR life-review).ab,ti
6. (electroconvulsive therapy OR electro-convulsive therapy OR Transcranial Magnetic Stimulation OR Transcranial Direct Current Stimulation OR light therapy).ab,ti
7. #1 OR #2 OR #3 OR #4 OR #5 OR #6
8. *Depressive Disorder, Major/ OR (major adj (depressive OR depression)).ab,ti
9. *Aged/ OR *"Aged, 80 and over"/ OR (elder* OR (older adj (person* OR people OR adult*)) OR (late adj life) OR geriatric).ab,ti
10. *Randomized Controlled Trial/ OR (Randomized Controlled Trial).pt OR *Random Allocation/
11. (singleblind* OR doubleblind* OR trebleblind* OR tripleblind*).ab,ti
12. (single* OR doubl* OR trebl* OR tripl*) adj5 blind*).ab,ti
13. (random*).ab,ti
14. (randomized OR randomised OR (random* adj (assigned OR allocated OR assignment OR allocation))).ab,ti
15. #10 OR ((#11 OR #12) AND #13) OR #14
16. #7 AND #8 AND #9 AND #15

Study selection

All potential articles will be retrieved and organized in a data management software (Endnote software, Thomson Reuters). After removing duplicate records, two reviewers will independently screen through the titles and abstracts to retain eligible articles. The first 10%

For peer review only

For peer review only - http://bmjopen.bmj.com/site/about/guidelines.xhtml

of these titles and abstracts will be subjected to a calibration exercise between the two reviewers to ensure mutual agreement.

After completing the screening phase, articles that are deemed as relevant by at least one of the reviewers will be subjected to full-text review. The two reviewers will independently confirm the eligibility of these articles based on the full texts. The first 10% of these full texts will again undergo a calibration exercise by the two reviewers. After the full-text review, the included articles will be used for qualitative synthesis. The chance-corrected agreement between the two reviewers will be assessed using Cohen's Kappa (κ).

At any point during study selection, the reasons for excluding specific articles will be recorded. Moreover, any disagreements between the two reviewers will be resolved by discussion with a third reviewer.

Data extraction

Data from the selected studies will be extracted by two reviewers independently, and disagreements between the reviewers will be resolved by discussion with a third reviewer. The extracted data will include the following information:

1. Study identification (first author, year of publication, geographic location)
2. Study characteristics (study setting, study design, inclusion criteria, diagnostic criteria of major depression, sample size)
3. Participant characteristics (age, gender, education, number of comorbidities, Mini Mental State Examination score, baseline depression score, depression scale, duration of the current episode of major depression)

For peer review only - http://bmjopen.bmj.com/site/about/guidelines.xhtml

4. Characteristics of intervention and comparator (description, treatment dose/intensity, treatment duration, depression score, all-cause discontinuation)

The original authors of the RCTs will be contacted when the required data are not available in the published article.

Assessment of risk of bias

The risk of bias for each study will be assessed independently by two reviewers using the Cochrane revised tool for Risk of Bias (RoB 2.0),[26] focusing on biases related to five key domains: randomization process, deviations from intended interventions, missing outcome data, measurement of the outcome and selection of the reported result. Each domain will receive a judgement on the risk of bias (high, low or some concerns) and an overall risk of bias will be assigned based on the judgements from the five domains. Any disagreements between the two reviewers will be resolved by discussion with a third reviewer.

Outcome measures

Our primary outcomes are the efficacy and the acceptability of interventions. The efficacy will be based on the difference in depression scores between the intervention and comparator upon the completion of intervention (we will give preference to the primary timepoint predefined in the original study), computed as standardized mean difference (SMD) for each RCT. The acceptability will be assessed by the relative risk (RR) of all-cause discontinuation of the intervention. Each intervention will only be grouped by its generic name for pharmacological interventions (such as mirtazapine, citalopram, quetiapine, valproate or

For peer review only - http://bmjopen.bmj.com/site/about/guidelines.xhtml

lithium) or by its known modality for non-pharmacological interventions (such as cognitive behavioural therapy, problem solving therapy, or transcranial magnetic stimulation). We will not categorize the interventions further in our analyses of the outcome measures. In the event that the active arm of a RCT involves combinations of interventions, it will be reported as the respective combinations (such as citalopram–cognitive behavioural therapy combination, or mirtazapine–quetiapine–problem solving therapy combination).

Statistical analysis

We will first conduct pairwise meta-analysis with the random-effects model (DerSimonian and Laird method)[27] provided there are at least two included studies for each pairwise comparison. We will use the I^2 statistic and the Q test to assess heterogeneity in each pairwise meta-analysis. In the presence of substantial heterogeneity (I^2>50%)[28] in a particular intervention, we will consider sub-grouping the intervention by its dose/intensity and duration, and use the subgroups of that intervention in the subsequent network meta-analyses.

We will then conduct the network meta-analyses within a Bayesian framework using the Markov Chains Monte Carlo method. Bayesian analysis provides probabilistic distributions of our estimates-of-interest through large number of simulations, and hence produces results which have more intuitive interpretations. For example, Bayesian analysis generates the 95% credible interval which can be accurately interpreted as the range containing 95% of the estimates (based on the simulations). In the Bayesian analysis, we will run four Markov chains simultaneously with different arbitrarily chosen initial values and with non-informative priors. Each chain will have at least 10,000 simulations and at least the first

For peer review only - http://bmjopen.bmj.com/site/about/guidelines.xhtml

2,500 simulations will be discarded as burn-in. Convergence of the simulations will be assessed with the trace plots, kernel density plots and Gelman-Rubin-Brooks plots.

We will employ both fixed-effects and random-effects models in the Bayesian analyses, and will choose the final models based on the deviance information criterion (DIC). While there is no rule-of-thumb on what constitute significant improvements in DIC, we can take reference from the guideline commonly used in the analogous Akaike Information Criteria:[29] values which are lesser by at least 10 points indicate significantly better model-fit and parsimony. Hence, results from the random-effects model will be used if the random-effects model has DIC which is smaller by at least 10 points compared to the fixed-effect model. We will also compare the complexity of model between the fixed-effects and random-effects models using pD (an indicator which has higher value when a model is more complex), with preference for models which are more parsimonious (less complex). The global heterogeneity will be assessed with I^2 statistic. A common heterogeneity parameter will be assumed in the random-effects model. Inconsistency between direct and indirect sources of evidence will be statistically assessed using the node-splitting method,[30 31] which generates a p-value for the difference between direct and indirect estimates in each closed-loop in the network (p-values of <0.05 indicates the presence of inconsistency between direct and indirect estimates in a particular closed-loop).

We will estimate the ranking probabilities for all interventions and show the results graphically in the form of rankograms and cumulative ranking probability plots. The hierarchy of interventions will be summarized as surface under the cumulative ranking curve (SUCRA) and presented in a scatterplot. SUCRAs have possible values ranging from 0% to

For peer review only - http://bmjopen.bmj.com/site/about/guidelines.xhtml

100%, with higher values indicating better efficacy or acceptability. Publication bias will be assessed with comparison-adjusted funnel plot.[32 33]

We will conduct meta-regression analyses to determine whether the results of our network meta-analyses will be affected by the following study-level covariates: sample size, study duration, inclusion criteria, study setting, study design and risk of bias. A covariate is considered as a significant moderator if the 95% credible interval of its beta coefficient in meta-regression does not include the value of zero. If a significant moderator is found, further subgroup analyses will then be conducted to assess the effect of this moderator.

The pairwise meta-analyses will be conducted with STATA (version 14). The network meta-analyses will be conducted using JAGS (version 4.2.0), through the GeMTC package of R (version 3.3.1). The "Network Graphs" package in Stata statistical software (version 14.0) will also used to produce some of the figures in this study, such as the network plots, rankograms, cumulative ranking probability plots and comparison-adjusted funnel plots. [32 34]

Assessment of quality of evidence

We will use the Grading of Recommendations Assessment, Development and Evaluation (GRADE) approach to report the quality of evidence on efficacy and acceptability of interventions for major depression in older persons. Based on five key domains (methodology quality, directness of evidence, heterogeneity, precision of effect estimates and risk of publication bias), we will classify the quality of evidence in one of four levels – high, moderate, low and very low.[35]

For peer review only - http://bmjopen.bmj.com/site/about/guidelines.xhtml

LIMITATIONS

Several limitations of this study should be noted. First, there can possibly be heterogeneity in the dose/intensity and the duration of each intervention, which may limit the interpretation of the meta-analysis. To address this potential limitation, we will first conduct pairwise meta-analyses to evaluate the amount of heterogeneity using the I^2 statistic and the Q test. In the presence of substantial heterogeneity (I^2>50%)[28] in a particular intervention, we will consider sub-grouping the intervention by its dose/intensity and duration, and use the more homogeneous subgroups of that intervention in the subsequent network meta-analyses. In the network meta-analyses, we will also evaluate for inconsistency between direct and indirect estimates using node-splitting method, and evaluate for heterogeneity using meta-regression and subgroup analyses. Second, we will exclude non-English and non-peer reviewed publications (such as conference proceedings and letters), which may potentially raise the concern of publication bias. The exclusion of non-peer reviewed publications is related to our intention of including only higher-quality evidence. Regardless, we will monitor the impact of such decision and any possible publication bias using comparison-adjusted funnel plot. Third, we will use all-cause discontinuation as a crude composite measure of treatment acceptability. All-cause discontinuation was chosen (instead of discontinuation due to specific reasons) because this information is more readily available in almost all RCTs, especially among non-pharmacological RCTs where it can be more challenging to clearly attribute the cause of discontinuation to specific reasons such as adverse effects. Hence, the use of all-cause discontinuation will allow us to compare the acceptability of both pharmacological and non-pharmacological interventions within the same model in network meta-analysis.

For peer review only - http://bmjopen.bmj.com/site/about/guidelines.xhtml

ETHICS AND DISSEMINATION

This systematic review will provide the consolidated evidence to inform clinicians on the best choice of intervention, from among the many available options, to address major depression in older persons. This systematic review will be reported in accordance with the recommendations of PRISMA statement for network meta-analyses.[36] It is expected to be completed by January 2020, and the results will be disseminated through conference presentations and publications in peer-reviewed journal.

CONTRIBUTORS

TML conceived the idea for this systematic review, developed the initial methodology, wrote the first draft and act as the guarantor of the protocol. CSL provided critical feedback on the search strategy, methodology and manuscript. All authors approved the final version of the manuscript.

FUNDING

TML was supported by research grants under the Singapore Ministry of Health's National Medical Research Council (Grant No.: NMRC/Fellowship/0030/2016 and NMRC/CSSSP/0014/2017). The funding source had no involvement in any part of the project.

COMPETING INTERESTS

For peer review only - http://bmjopen.bmj.com/site/about/guidelines.xhtml

1

2 None declared.

For peer review only - http://bmjopen.bmj.com/site/about/guidelines.xhtml

REFERENCES

1. World Health Organization. The global burden of disease: 2004 update. Switzerland: World Health Organization.

2. World Health Organization. Global Health Estimates 2015: Disease burden by Cause, Age, Sex, by Country and by Region, 2000-2015. Geneva, 2016.

3. Taylor WD. Clinical practice. Depression in the elderly. *The New England journal of medicine* 2014;371(13):1228-36. doi: 10.1056/NEJMcp1402180 [published Online First: 2014/09/25]

4. Blazer DG. Depression in late life: review and commentary. *The journals of gerontology Series A, Biological sciences and medical sciences* 2003;58(3):249-65. [published Online First: 2003/03/14]

5. Volkert J, Schulz H, Härter M, et al. The prevalence of mental disorders in older people in Western countries – a meta-analysis. *Ageing Research Reviews* 2013;12(1):339-53. doi: http://dx.doi.org/10.1016/j.arr.2012.09.004

6. Lyness JM, Caine ED, King DA, et al. Psychiatric Disorders in Older Primary Care Patients. *Journal of general internal medicine* 1999;14(4):249-54. doi: 10.1046/j.1525-1497.1999.00326.x

7. Jackson JC, Pandharipande PP, Girard TD, et al. Depression, Posttraumatic Stress Disorder, and Functional Disability in Survivors of Critical Illness: results from the BRAIN ICU (Bringing to light the Risk Factors And Incidence of Neuropsychological dysfunction in ICU survivors) Investigation: A Longitudinal Cohort Study. *The lancet Respiratory medicine* 2014;2(5):369-79. doi: 10.1016/S2213-2600(14)70051-7

For peer review only - http://bmjopen.bmj.com/site/about/guidelines.xhtml

8. Gan Y, Gong Y, Tong X, et al. Depression and the risk of coronary heart disease: a meta-analysis of prospective cohort studies. *BMC psychiatry* 2014;14:371. doi: 10.1186/s12888-014-0371-z [published Online First: 2014/12/30]

9. Pan A, Sun Q, Okereke OI, et al. Depression and risk of stroke morbidity and mortality: a meta-analysis and systematic review. *Jama* 2011;306(11):1241-9. doi: 10.1001/jama.2011.1282 [published Online First: 2011/09/22]

10. Cuijpers P, Vogelzangs N, Twisk J, et al. Comprehensive meta-analysis of excess mortality in depression in the general community versus patients with specific illnesses. *The American journal of psychiatry* 2014;171(4):453-62. doi: 10.1176/appi.ajp.2013.13030325 [published Online First: 2014/01/18]

11. Tham A, Jonsson U, Andersson G, et al. Efficacy and tolerability of antidepressants in people aged 65 years or older with major depressive disorder – A systematic review and a meta-analysis. *Journal of affective disorders* 2016;205:1-12. doi: http://dx.doi.org/10.1016/j.jad.2016.06.013

12. Nelson JC, Delucchi K, Schneider LS. Efficacy of second generation antidepressants in late-life depression: a meta-analysis of the evidence. *The American journal of geriatric psychiatry : official journal of the American Association for Geriatric Psychiatry* 2008;16(7):558-67. doi: 10.1097/JGP.0b013e3181693288 [published Online First: 2008/07/02]

13. Thorlund K, Druyts E, Wu P, et al. Comparative efficacy and safety of selective serotonin reuptake inhibitors and serotonin-norepinephrine reuptake inhibitors in older adults: a network meta-analysis. *Journal of the American Geriatrics Society* 2015;63(5):1002-9. doi: 10.1111/jgs.13395 [published Online First: 2015/05/07]

14. Tedeschini E, Levkovitz Y, Iovieno N, et al. Efficacy of antidepressants for late-life depression: a meta-analysis and meta-regression of placebo-controlled randomized

For peer review only - http://bmjopen.bmj.com/site/about/guidelines.xhtml

trials. *The Journal of clinical psychiatry* 2011;72(12):1660-8. doi: 10.4088/JCP.10r06531 [published Online First: 2012/01/17]

15. Gould RL, Coulson MC, Howard RJ. Cognitive behavioral therapy for depression in older people: a meta-analysis and meta-regression of randomized controlled trials. *Journal of the American Geriatrics Society* 2012;60(10):1817-30. doi: 10.1111/j.1532-5415.2012.04166.x [published Online First: 2012/09/26]

16. Kirkham JG, Choi N, Seitz DP. Meta-analysis of problem solving therapy for the treatment of major depressive disorder in older adults. *International journal of geriatric psychiatry* 2016;31(5):526-35. doi: 10.1002/gps.4358 [published Online First: 2015/10/06]

17. Cuijpers P, van Straten A, Smit F. Psychological treatment of late-life depression: a meta-analysis of randomized controlled trials. *International journal of geriatric psychiatry* 2006;21(12):1139-49. doi: 10.1002/gps.1620 [published Online First: 2006/09/07]

18. Cuijpers P, Karyotaki E, Pot AM, et al. Managing depression in older age: psychological interventions. *Maturitas* 2014;79(2):160-69. doi: 10.1016/j.maturitas.2014.05.027

19. Linde K, Sigterman K, Kriston L, et al. Effectiveness of Psychological Treatments for Depressive Disorders in Primary Care: Systematic Review and Meta-Analysis. *The Annals of Family Medicine* 2015;13(1):56-68. doi: 10.1370/afm.1719

20. Holvast F, Massoudi B, Oude Voshaar RC, et al. Non-pharmacological treatment for depressed older patients in primary care: A systematic review and meta-analysis. *PloS one* 2017;12(9):e0184666. doi: 10.1371/journal.pone.0184666

21. Apostolo J, Bobrowicz-Campos E, Rodrigues M, et al. The effectiveness of non-pharmacological interventions in older adults with depressive disorders: A systematic review. *International journal of nursing studies* 2016;58:59-70. doi: 10.1016/j.ijnurstu.2016.02.006 [published Online First: 2016/04/19]

For peer review only - http://bmjopen.bmj.com/site/about/guidelines.xhtml

22. Sukhato K, Lotrakul M, Dellow A, et al. Efficacy of home-based non-pharmacological interventions for treating depression: a systematic review and network meta-analysis of randomised controlled trials. *BMJ open* 2017;7(7)

23. Tonin FS, Rotta I, Mendes AM, et al. Network meta-analysis: a technique to gather evidence from direct and indirect comparisons. *Pharmacy practice* 2017;15(1):943. doi: 10.18549/PharmPract.2017.01.943 [published Online First: 2017/05/16]

24. Moher D, Shamseer L, Clarke M, et al. Preferred reporting items for systematic review and meta-analysis protocols (PRISMA-P) 2015 statement. *Systematic reviews* 2015;4:1. doi: 10.1186/2046-4053-4-1 [published Online First: 2015/01/03]

25. Shamseer L, Moher D, Clarke M, et al. Preferred reporting items for systematic review and meta-analysis protocols (PRISMA-P) 2015: elaboration and explanation. *BMJ (Clinical research ed)* 2015;349:g7647. doi: 10.1136/bmj.g7647 [published Online First: 2015/01/04]

26. Higgins JPT, Sterne JAC, Savović J, et al. A revised tool for assessing risk of bias in randomized trials. In: Chandler J, McKenzie J, Boutron I, et al., eds. Cochrane Methods Cochrane Database of Systematic Reviews 2016, Issue 10 (Suppl 1).

27. DerSimonian R, Laird N. Meta-analysis in clinical trials. *Controlled clinical trials* 1986;7(3):177-88. [published Online First: 1986/09/01]

28. Higgins JP, Thompson SG, Deeks JJ, et al. Measuring inconsistency in meta-analyses. *BMJ (Clinical research ed)* 2003;327(7414):557-60. doi: 10.1136/bmj.327.7414.557 [published Online First: 2003/09/06]

29. Burnham KP, Anderson DR. Model Selection and Multimodel Inference: A Practical Information-Theoretic Approach: Springer New York 2003.

For peer review only

For peer review only - http://bmjopen.bmj.com/site/about/guidelines.xhtml

30. Dias S, Welton NJ, Caldwell DM, et al. Checking consistency in mixed treatment comparison meta-analysis. *Statistics in medicine* 2010;29(7-8):932-44. doi: 10.1002/sim.3767 [published Online First: 2010/03/10]

31. van Valkenhoef G, Dias S, Ades AE, et al. Automated generation of node-splitting models for assessment of inconsistency in network meta-analysis. *Research synthesis methods* 2016;7(1):80-93. doi: 10.1002/jrsm.1167 [published Online First: 2015/10/16]

32. Chaimani A, Higgins JP, Mavridis D, et al. Graphical tools for network meta-analysis in STATA. *PloS one* 2013;8(10):e76654. doi: 10.1371/journal.pone.0076654 [published Online First: 2013/10/08]

33. Peters JL, Sutton AJ, Jones DR, et al. Assessing publication bias in meta-analyses in the presence of between-study heterogeneity. *Journal of the Royal Statistical Society: Series A (Statistics in Society)* 2010;173(3):575-91. doi: 10.1111/j.1467-985X.2009.00629.x

34. Chaimani A, Salanti G. Visualizing assumptions and results in network meta-analysis: The network graphs package. *Stata Journal* 2015;15(4):905-50.

35. Schunemann H, Brozek J, Guyatt G, et al. GRADE handbook for grading quality of evidence and strength of recommendation 2013. Available from: http://gdt.guidelinedevelopment.org/app/ (assessed 22 Nov 2016).

36. Hutton B, Salanti G, Caldwell DM, et al. The PRISMA extension statement for reporting of systematic reviews incorporating network meta-analyses of health care interventions: checklist and explanations. *Annals of internal medicine* 2015;162(11):777-84. doi: 10.7326/m14-2385 [published Online First: 2015/06/02]

For peer review only - http://bmjopen.bmj.com/site/about/guidelines.xhtml

PRISMA-P (Preferred Reporting Items for Systematic review and Meta-Analysis Protocols) 2015 checklist: recommended items to address in a systematic review protocol*

Section and topic	Item No	Checklist item	Page (Line)
**ADMINISTRATIVE INFORMATION**			
Title:			
Identification	1a	Identify the report as a protocol of a systematic review	1 (2)
Update	1b	If the protocol is for an update of a previous systematic review, identify as such	Not applicable
Registration	2	If registered, provide the name of the registry (such as PROSPERO) and registration number	3 (5)
Authors:			
Contact	3a	Provide name, institutional affiliation, e-mail address of all protocol authors; provide physical mailing address of corresponding author	1 (11)
Contributions	3b	Describe contributions of protocol authors and identify the guarantor of the review	Not applicable
Amendments	4	If the protocol represents an amendment of a previously completed or published protocol, identify as such and list changes; otherwise, state plan for documenting important protocol amendments	Not applicable
Support:			
Sources	5a	Indicate sources of financial or other support for the review	17 (10)
Sponsor	5b	Provide name for the review funder and/or sponsor	Not applicable
Role of sponsor or funder	5c	Describe roles of funder(s), sponsor(s), and/or institution(s), if any, in developing the protocol	17 (12)
**INTRODUCTION**			
Rationale	6	Describe the rationale for the review in the context of what is already known	5 (5)
Objectives	7	Provide an explicit statement of the question(s) the review will address with reference to participants, interventions, comparators, and outcomes (PICO)	5 (24)
**METHODS**			
Eligibility criteria	8	Specify the study characteristics (such as PICO, study design, setting, time frame) and report characteristics (such as years considered, language, publication status) to be used as criteria for eligibility for the review	6 (14)
Information sources	9	Describe all intended information sources (such as electronic databases, contact with study authors, trial registers or other grey literature sources) with planned dates of coverage	9 (8)
Search strategy	10	Present draft of search strategy to be used for at least one electronic database, including planned limits, such that it could be repeated	9 (11)

For peer review only

Study records:			
Data management	11a	Describe the mechanism(s) that will be used to manage records and data throughout the review	10 (4)
Selection process	11b	State the process that will be used for selecting studies (such as two independent reviewers) through each phase of the review (that is, screening, eligibility and inclusion in meta-analysis)	10 (5)
Data collection process	11c	Describe planned method of extracting data from reports (such as piloting forms, done independently, in duplicate), any processes for obtaining and confirming data from investigators	11 (14)
Data items	12	List and define all variables for which data will be sought (such as PICO items, funding sources), any pre-planned data assumptions and simplifications	11 (16)
Outcomes and prioritization	13	List and define all outcomes for which data will be sought, including prioritization of main and additional outcomes, with rationale	12 (16)
Risk of bias in individual studies	14	Describe anticipated methods for assessing risk of bias of individual studies, including whether this will be done at the outcome or study level, or both; state how this information will be used in data synthesis	12 (6)
Data synthesis	15a	Describe criteria under which study data will be quantitatively synthesised	Not applicable
	15b	If data are appropriate for quantitative synthesis, describe planned summary measures, methods of handling data and methods of combining data from studies, including any planned exploration of consistency (such as I^2, Kendall's τ)	13 (7)
	15c	Describe any proposed additional analyses (such as sensitivity or subgroup analyses, meta-regression)	13 (14)
	15d	If quantitative synthesis is not appropriate, describe the type of summary planned	Not applicable
Meta-bias(es)	16	Specify any planned assessment of meta-bias(es) (such as publication bias across studies, selective reporting within studies)	14 (22)
Confidence in cumulative evidence	17	Describe how the strength of the body of evidence will be assessed (such as GRADE)	15 16)

*** It is strongly recommended that this checklist be read in conjunction with the PRISMA-P Explanation and Elaboration (cite when available) for important clarification on the items. Amendments to a review protocol should be tracked and dated. The copyright for PRISMA-P (including checklist) is held by the PRISMA-P Group and is distributed under a Creative Commons Attribution Licence 4.0.**

From: Shamseer L, Moher D, Clarke M, Ghersi D, Liberati A, Petticrew M, Shekelle P, Stewart L, PRISMA-P Group. Preferred reporting items for systematic review and meta-analysis protocols (PRISMA-P) 2015: elaboration and explanation. BMJ. 2015 Jan 2;349(jan02 1):g7647.

For peer review only

BMJ Open

Comparative efficacy and acceptability of interventions for major depression in older persons: protocol for Bayesian network meta-analysis

Journal:	*BMJ Open*
Manuscript ID	bmjopen-2017-019819.R2
Article Type:	Protocol
Date Submitted by the Author:	23-Nov-2017
Complete List of Authors:	Liew, Tau Ming; Institute of Mental Health, Department of Geriatric Psychiatry<br>Lee, Cia Sin; SingHealth Polyclinics, Sengkang Polyclinic
**Primary Subject Heading**:	Mental health
Secondary Subject Heading:	Geriatric medicine
Keywords:	major depression, older person, efficacy, acceptability, network meta-analysis

SCHOLARONE™ Manuscripts

For peer review only

Comparative efficacy and acceptability of interventions for major depression in older persons: protocol for Bayesian network meta-analysis

Tau Ming Liew[1, 2], Cia Sin Lee[3]

[1]Department of Geriatric Psychiatry, Institute of Mental Health, Singapore

[2]Saw Swee Hock School of Public Health, National University of Singapore

[3]SingHealth Polyclinics, Singapore

Correspondence to

Tau Ming Liew;

tau_ming_liew@imh.com.sg

Department of Geriatric Psychiatry, Institute of Mental Health,

10 Buangkok View, Singapore 539747.

Keywords: major depression; older person; efficacy; acceptability; network meta-analysis

Number of words (Abstract): 290

Number of words (main text): 2,946

Number of references: 36

Number of tables or figures: 1

For peer review only - http://bmjopen.bmj.com/site/about/guidelines.xhtml

ABSTRACT

Introduction: Major depression is a leading cause of disability, and has been associated with adverse effects in older persons. While many pharmacological and non-pharmacological interventions have been shown to be effective to address major depression in older persons, there has not been a meta-analysis that consolidates all the available interventions and compare the relative benefits of these available interventions. In this study, we aim to conduct a systematic review and network meta-analysis to compare the efficacy and acceptability of all the known pharmacological and non-pharmacological interventions for major depression in older persons.

Methods and analysis: We will search MEDLINE, Embase, PsycINFO, Cumulative Index to Nursing and Allied Health, Cochrane Central Register of Controlled Trials and references of other review articles for articles related to the keywords of 'randomized trial', 'major depression', 'older persons' and 'treatments'. Two reviewers will independently select the eligible articles. For each included article, the two reviewers will independently extract the data and assess the risk of bias using the Cochrane revised tool for Risk of Bias. Bayesian network meta-analyses will be conducted to pool the depression scores (based on standardized mean difference) and the all-cause discontinuation across all included studies. The ranking probabilities for all interventions will be estimated and the hierarchy of each interventions will be summarized as surface under the cumulative ranking curve (SUCRA). Meta-regression and sub-group analyses will also be performed to evaluate the effect of study-level covariates. The quality of the evidence will be assessed using the Grading of Recommendations Assessment, Development and Evaluation (GRADE) approach.

Ethics and dissemination: The results will be disseminated through conference presentations and peer-reviewed publications. They will provide the consolidated evidence to

For peer review only

inform clinicians on the best choice of intervention to address major depression in older persons.

Trial registration number: International Prospective Register for Systematic Reviews (PROSPERO) number CRD42017075756.

For peer review only

For peer review only - http://bmjopen.bmj.com/site/about/guidelines.xhtml

STRENGTHS AND LIMITATIONS OF THIS STUDY

- This systematic review and meta-analysis will provide a comprehensive summary on the efficacy and acceptability of all available interventions for major depression in older persons.
- The results will provide the highest level of evidence to inform clinicians on the best choice of treatment, from among the many available pharmacological and non-pharmacological interventions.
- This protocol has been developed in accordance with the Preferred Reporting Items for Systematic Review and Meta-analysis Protocols (PRISMA-P) statement and has been registered with PROSPERO.
- The overall quality of evidence will be assessed using the Grading of Recommendations Assessment, Development and Evaluation (GRADE) approach.
- This systematic review will be limited to studies which are reported in English language and have been peer-reviewed.

For peer review only - http://bmjopen.bmj.com/site/about/guidelines.xhtml

INTRODUCTION

Rationale

Major depression has been identified by the World Health Organization as one of the leading cause of disability globally.[1 2] In older persons, its prevalence rates rise with the increase in medical comorbidities,[3] with reported rates of up to 5% in community-dwelling older persons,[3-5] 5 to 10% in primary care[3 6] and as high as 37% after critical care hospitalizations.[3 7] Major depression has a significant impact on the older populations and has been linked to higher risk of suicide,[4] myocardial infarction,[8] stroke,[9] all-cause mortality[4 10] and increasing health services utilization.[4]

Many of the interventions for major depression in older persons have had recent meta-analyses confirming their efficacy when compared to control groups. These include antidepressants,[11-14] cognitive behavioural therapy,[15] problem solving therapy,[16] psychological interventions in general,[17-19] and the various forms of non-pharmacological interventions.[20-22] However, none of the meta-analyses had compared all the pharmacological and non-pharmacological interventions together to demonstrate the relative benefits of each intervention. It is unknown whether the different types of pharmacological and non-pharmacological interventions have comparable efficacy and are equally suitable for older persons with major depression.

Objectives

For peer review only - http://bmjopen.bmj.com/site/about/guidelines.xhtml

In this study, we aim to conduct a systematic review and network meta-analysis to compare the efficacy and acceptability of all the available pharmacological and non-pharmacological interventions for major depression in older persons. The use of network meta-analysis allows us to pool the evidence on various interventions and rank their benefits relative to each other.[23] It also allows us to conduct indirect comparison of the different interventions, even when there is no direct evidence in the literature to allow head-to-head comparisons.

METHODS AND ANALYSIS

This protocol is developed in accordance with the Preferred Reporting Items for Systematic Review and Meta-analysis (PRISMA) statement.[24 25] It has also been registered with the International Prospective Register of Systematic Reviews (PROSPERO) (registration number CRD42017075756).

Eligibility criteria

Participants and settings

We will include studies which recruited participants who were:

- 60 years old and above;
- diagnosed with major depression based on formal criteria by the Diagnostic and Statistical Manual of Mental Disorders (DSM) or International Classification of Diseases (ICD); and

For peer review only - http://bmjopen.bmj.com/site/about/guidelines.xhtml

- having a current episode of major depression (that is, the participants were symptomatic and not in remission at the point of recruitment; and the intervention was not intended primarily for the prevention of future relapses).

We will exclude studies which recruited participants with treatment-resistant depression, subthreshold depression, bipolar depression, depression in dementia or psychotic depression. We will not include maintenance studies for major depression as such studies primarily focused on the prevention of relapses in participants who had been asymptomatic or in remission at the point of recruitment.

Interventions

We will include studies with pharmacological interventions, including but not limited to: [26 27]

- Antidepressants such as citalopram, sertraline, venlafaxine or mirtazapine;
- Antipsychotics such as risperidone, quetiapine, olanzapine or aripiprazole;
- Mood-stabilizers such as valproate, carbamazepine, lithium or gabapentin.

We will include studies with non-pharmacological interventions, including but not limited to: [28-30]

- Psychological interventions such as cognitive behavioural therapy, problem solving therapy, interpersonal therapy, family interventions or psychodynamic therapy;
- Procedural interventions such as electroconvulsive therapy, transcranial magnetic stimulation, transcranial direct-current stimulation or bright light therapy.

For peer review only - http://bmjopen.bmj.com/site/about/guidelines.xhtml

We will also include studies which reported on combinations of any of these pharmacological and non-pharmacological interventions.

We will exclude studies which focused primarily on health service models of care but were not related to any modality of intervention, such as studies which evaluated the effectiveness of home treatment, training of general practitioners, multidisciplinary approach or stepped-care approach.

Comparators

We will accept control conditions such as placebo intervention, waiting-list, treatment as usual, as well as no intervention. We will also include studies with active comparators such as those which compare between two different interventions within the same studies.

Outcomes

We will only include a study if it reports the depression scores or the all-cause discontinuation in each study arm following intervention.

Study designs and publication types

We will only include randomized controlled trials (RCTs) which aimed to demonstrate the superiority of a treatment to another (also known as superiority trials), and will not include equivalence or non-inferiority trials. The following study designs or publication types will also be excluded: qualitative studies, observational studies, meta-analyses, case reports, case

For peer review only - http://bmjopen.bmj.com/site/about/guidelines.xhtml

series, ecological studies and policy papers. We intend to include only higher-quality evidence and hence will exclude non-randomized trials and publications which were not peer-reviewed (such as conference proceedings, letters and comments).

Language and time frame

We will only include studies which are reported in the English language. Apart from that, we do not impose any time restriction to the publication year of the studies. The search of databases will be conducted in January 2018.

Information sources and search strategy

We will search MEDLINE, Embase, PsycINFO, Cumulative Index to Nursing and Allied Health (CINAHL) and Cochrane Central Register of Controlled Trials (CENTRAL) for original articles related to the keywords of 'randomized trial', 'major depression', 'older persons' and 'treatments'. Our search strategy for MEDLINE is shown in Box 1. Similar search strategies will be used for the other databases. Additionally, we will also hand-search the references of review articles related to the topic to retrieve relevant articles which are not captured through our search of the electronic databases. We will examine the full text of the relevant articles and include the respective articles if they meet our eligibility criteria.

**Box 1. Search strategy for MEDLINE (via Ovid interface)**
1. *Therapeutics/ OR *Drug Therapy/ OR *Psychotropic Drugs/ OR *Antidepressive Agents/ OR *Antipsychotic Agents/ OR *Antimanic Agents/ OR *Anticonvulsants/ OR *Psychotherapy/ OR *Electroconvulsive Therapy/ OR *Transcranial Magnetic Stimulation/ OR *Transcranial Direct Current Stimulation/ OR *Phototherapy/
2. (antidepressant* OR "selective serotonin reuptake inhibitor" OR SSRI OR citalopram OR fluoxetine OR paroxetine OR sertraline OR escitalopram OR fluvoxamine OR "serotonin and epinephrine reuptake inhibitor" OR "serotonin epinephrine reuptake inhibitor" OR SNRI OR

For peer review only - http://bmjopen.bmj.com/site/about/guidelines.xhtml

venlafaxine OR desvenlafaxine OR duloxetine OR milnacipran OR reboxetine OR bupropion OR “noradrenergic and specific serotonergic antidepressant” OR NaSSA OR mirtazapine OR TCA OR tricyclic OR amersergide OR amineptine OR amitriptyline OR amoxapine OR butriptyline OR chlorpoxiten OR clomipramine OR clorimipramine OR demexiptiline OR desipramine OR dibenzipin OR dothiepin OR doxepin OR imipramine OR lofepramine OR melitracen OR metapramine OR nortriptyline OR noxiptiline OR opipramol OR protriptyline OR quinupramine OR trimipramine OR tianeptine OR trazodone OR nefazodone OR agomelatine).ab,ti

3. (antipsychotic* OR haloperidol OR trifluoperazine OR benperidol OR chlorprothixene OR flupenthixol OR clopenthixol OR chlorpromazine OR prochlorperazine OR sulpiride OR periciazine OR perphenazine OR pimozide OR promazine OR fluspirilene OR methotrimeprazine OR risperidone OR paliperidone OR quetiapine OR olanzapine OR amisulpride OR amisulpiride OR aripiprazole OR clozapine OR sertindole OR zotepine).ab,ti
4. ((mood adj stabili*) OR (antimanic adj (agent* OR drug*)) OR anticonvuls* OR anti convuls* OR carbamazepine OR ethosuximide OR gabapentin OR lacosamide OR lamotrigine OR levetiracetam OR lithium OR oxcarbazepine OR phenobarbital OR phenytoin OR pregabalin OR rufinamide OR tiagabine OR topiramate OR valproic acid OR valproate OR verapamil OR vigabatrin OR zonisamide).ab,ti
5. (psychotherap* OR therap* OR (cognitive adj behavio* adj therapy) OR “cognitive therapy” OR behavio* adj therapy OR “problem solving therapy” OR “problem-solving therapy” OR “interpersonal therapy” OR “inter-personal therapy” OR (family adj (therapy OR intervention)) OR psychodynamic OR psychoanalytic OR bibliotherapy OR mindful* OR (group adj (therapy OR intervention)) OR emotion-focused OR “emotion focused” OR reminiscen* OR “life review” OR life-review).ab,ti
6. (“electroconvulsive therapy” OR “electro-convulsive therapy” OR “Transcranial Magnetic Stimulation” OR “Transcranial Direct Current Stimulation” OR “light therapy”).ab,ti
7. #1 OR #2 OR #3 OR #4 OR #5 OR #6
8. *Depressive Disorder, Major/ OR (major adj (depressive OR depression)).ab,ti
9. *Aged/ OR *"Aged, 80 and over"/ OR (elder* OR (older adj (person* OR people OR adult*)) OR (late adj life) OR geriatric).ab,ti
10. *Randomized Controlled Trial/ OR (Randomized Controlled Trial).pt OR *Random Allocation/
11. (randomized OR randomised OR (random* adj (assigned OR allocated OR assignment OR allocation))).ab,ti
12. #10 OR #11
13. #7 AND #8 AND #9 AND #12

Study selection

All potential articles will be retrieved and organized in a data management software (Endnote software, Thomson Reuters). After removing duplicate records, two reviewers will

For peer review only - http://bmjopen.bmj.com/site/about/guidelines.xhtml

independently screen through the titles and abstracts to retain eligible articles. The first 10% of these titles and abstracts will be subjected to a calibration exercise between the two reviewers to ensure mutual agreement.

After completing the screening phase, articles that are deemed as relevant by at least one of the reviewers will be subjected to full-text review. The two reviewers will independently confirm the eligibility of these articles based on the full texts. The first 10% of these full texts will again undergo a calibration exercise by the two reviewers. After the full-text review, the included articles will be used for qualitative synthesis. The chance-corrected agreement between the two reviewers will be assessed using Cohen's Kappa (κ).

At any point during study selection, the reasons for excluding specific articles will be recorded. Moreover, any disagreements between the two reviewers will be resolved by discussion with a third reviewer.

Data extraction

Data from the selected studies will be extracted by two reviewers independently, and disagreements between the reviewers will be resolved by discussion with a third reviewer. The extracted data will include the following information:

1. Study identification (first author, year of publication, geographic location)
2. Study characteristics (study setting, study design, inclusion criteria, diagnostic criteria of major depression, sample size)

For peer review only - http://bmjopen.bmj.com/site/about/guidelines.xhtml

3. Participant characteristics (age, gender, education, number of comorbidities, Mini Mental State Examination score, baseline depression score, depression scale, duration of the current episode of major depression)
4. Characteristics of intervention and comparator (description, treatment dose/intensity, treatment duration, depression score, all-cause discontinuation)

The original authors of the RCTs will be contacted when the required data are not available in the published article.

Assessment of risk of bias

The risk of bias for each study will be assessed independently by two reviewers using the Cochrane revised tool for Risk of Bias (RoB 2.0),[31] focusing on biases related to five key domains: randomization process, deviations from intended interventions, missing outcome data, measurement of the outcome and selection of the reported result. Each domain will receive a judgement on the risk of bias (high, low or some concerns) and an overall risk of bias will be assigned based on the judgements from the five domains. Any disagreements between the two reviewers will be resolved by discussion with a third reviewer.

Outcome measures

Our primary outcomes are the efficacy and the acceptability of interventions. The efficacy will be based on the difference in depression scores between the intervention and comparator upon the completion of intervention (we will give preference to the primary timepoint predefined in the original study), computed as standardized mean difference (SMD) for each

For peer review only - http://bmjopen.bmj.com/site/about/guidelines.xhtml

RCT. The acceptability will be assessed by the relative risk (RR) of all-cause discontinuation of the intervention. When the information is available, we will also capture a secondary outcome of discontinuation due to adverse effects of interventions and evaluate the RR of discontinuation due to adverse effects. Each intervention will only be grouped by its generic name for pharmacological interventions (such as citalopram, risperidone, or valproate) or by its known modality for non-pharmacological interventions (such as cognitive behavioural therapy, problem solving therapy, or electroconvulsive therapy). We will not categorize the interventions further in our analyses of the outcome measures. In the event that the active arm of a RCT involves combinations of interventions, it will be reported as the respective combinations (such as citalopram–cognitive behavioural therapy combination, or risperidone–problem solving therapy combination).

Statistical analysis

We will first conduct pairwise meta-analysis provided there are at least two included studies for each pairwise comparison. If there are at least five included studies, we will use the random effects model (DerSimonian and Laird method)[32] to pool the results because this model does not assume homogeneity among the pooled studies. If there are less than five included studies, the random effects model is imprecise in its estimations[33 34] and we will choose the fixed effect model (Mantel-Haenszel method)[35] instead. We will use the I^2 statistic and the Q test to assess heterogeneity in each pairwise meta-analysis. In the presence of substantial heterogeneity (I^2>50%)[36] in a particular intervention, we will consider sub-grouping the intervention by its dose/intensity and duration, and use the subgroups of that intervention in the subsequent network meta-analyses.

For peer review only - http://bmjopen.bmj.com/site/about/guidelines.xhtml

We will then conduct the network meta-analyses within a Bayesian framework using the Markov Chains Monte Carlo method. Bayesian analysis provides probabilistic distributions of our estimates-of-interest through large number of simulations, and hence produces results which have more intuitive interpretations. For example, Bayesian analysis generates the 95% credible interval which can be accurately interpreted as the range containing 95% of the estimates (based on the simulations). In the Bayesian analysis, we will run four Markov chains simultaneously with different arbitrarily chosen initial values and with non-informative priors. Each chain will have at least 10,000 simulations and at least the first 2,500 simulations will be discarded as burn-in. Convergence of the simulations will be assessed with the trace plots, kernel density plots and Gelman-Rubin-Brooks plots.

We will employ both fixed-effects and random-effects models in the Bayesian analyses, and will choose the final models based on the deviance information criterion (DIC). While there is no rule-of-thumb on what constitute significant improvements in DIC, we can take reference from the guideline commonly used in the analogous Akaike Information Criteria:[37] values which are lesser by at least 10 points indicate significantly better model-fit and parsimony. Hence, results from the random-effects model will be used if the random-effects model has DIC which is smaller by at least 10 points compared to the fixed-effect model. We will also compare the complexity of model between the fixed-effects and random-effects models using pD (an indicator which has higher value when a model is more complex), with preference for models which are more parsimonious (less complex). The global heterogeneity will be assessed with I^2 statistic. A common heterogeneity parameter will be assumed in the random-effects model. Inconsistency between direct and indirect sources of evidence will be statistically assessed using the node-splitting method,[38 39] which generates a p-value for the difference between direct and indirect estimates in each closed-loop in the

For peer review only - http://bmjopen.bmj.com/site/about/guidelines.xhtml

network (p-values of <0.05 indicates the presence of inconsistency between direct and indirect estimates in a particular closed-loop).

We will estimate the ranking probabilities for all interventions and show the results graphically in the form of rankograms and cumulative ranking probability plots. The hierarchy of interventions will be summarized as surface under the cumulative ranking curve (SUCRA) and presented in a scatterplot. SUCRAs have possible values ranging from 0% to 100%, with higher values indicating better efficacy or acceptability. Publication bias will be assessed with comparison-adjusted funnel plot.[40 41]

We will conduct meta-regression analyses to determine whether the results of our network meta-analyses will be affected by the following study-level covariates: sample size, study duration, inclusion criteria, study setting, study design and risk of bias. A covariate is considered as a significant moderator if the 95% credible interval of its beta coefficient in meta-regression does not include the value of zero. If a significant moderator is found, further subgroup analyses will then be conducted to assess the effect of this moderator.

The pairwise meta-analyses will be conducted with STATA (version 14). The network meta-analyses will be conducted using JAGS (version 4.2.0), through the GeMTC package of R (version 3.3.1). The "Network Graphs" package in Stata statistical software (version 14.0) will also used to produce some of the figures in this study, such as the network plots, rankograms, cumulative ranking probability plots and comparison-adjusted funnel plots. [40 42]

Assessment of quality of evidence

For peer review only - http://bmjopen.bmj.com/site/about/guidelines.xhtml

We will use the Grading of Recommendations Assessment, Development and Evaluation (GRADE) approach to report the quality of evidence on efficacy and acceptability of interventions for major depression in older persons. Based on five key domains (methodology quality, directness of evidence, heterogeneity, precision of effect estimates and risk of publication bias), we will classify the quality of evidence in one of four levels – high, moderate, low and very low.[43]

LIMITATIONS

Several limitations of this study should be noted. First, there can possibly be heterogeneity in the dose/intensity and the duration of each intervention, which may limit the interpretation of the meta-analysis. To address this potential limitation, we will first conduct pairwise meta-analyses to evaluate the amount of heterogeneity using the I^2 statistic and the Q test. In the presence of substantial heterogeneity (I^2>50%)[36] in a particular intervention, we will consider sub-grouping the intervention by its dose/intensity and duration, and use the more homogeneous subgroups of that intervention in the subsequent network meta-analyses. In the network meta-analyses, we will also evaluate for inconsistency between direct and indirect estimates using node-splitting method, and evaluate for heterogeneity using meta-regression and subgroup analyses. Second, we will exclude non-English and non-peer reviewed publications (such as conference proceedings and letters). The exclusion of non-peer reviewed publications is related to our intention of including only higher-quality evidence. Regardless, we will monitor the impact of such decision and any possible publication bias using comparison-adjusted funnel plot. Third, we will use all-cause discontinuation as a crude composite measure of treatment acceptability. All-cause discontinuation was chosen (instead of discontinuation due to specific reasons) because this information is more readily

For peer review only - http://bmjopen.bmj.com/site/about/guidelines.xhtml

available in almost all RCTs, especially among non-pharmacological RCTs where it can be more challenging to clearly attribute the cause of discontinuation to specific reasons such as adverse effects. Hence, the use of all-cause discontinuation will allow us to compare the acceptability of both pharmacological and non-pharmacological interventions within the same model in network meta-analysis.

ETHICS AND DISSEMINATION

This systematic review will provide the consolidated evidence to inform clinicians on the best choice of intervention, from among the many available options, to address major depression in older persons. This systematic review will be reported in accordance with the recommendations of PRISMA statement for network meta-analyses.[44] It is expected to be completed by January 2020, and the results will be disseminated through conference presentations and publications in peer-reviewed journal.

CONTRIBUTORS

TML conceived the idea for this systematic review, developed the initial methodology, wrote the first draft and act as the guarantor of the protocol. CSL provided critical feedback on the search strategy, methodology and manuscript. All authors approved the final version of the manuscript.

FUNDING

For peer review only - http://bmjopen.bmj.com/site/about/guidelines.xhtml

TML was supported by research grants under the Singapore Ministry of Health's National Medical Research Council (Grant No.: NMRC/Fellowship/0030/2016 and NMRC/CSSSP/0014/2017). The funding source had no involvement in any part of the project.

COMPETING INTERESTS

None declared.

For peer review only

REFERENCES

1. World Health Organization. The global burden of disease: 2004 update. Switzerland: World Health Organization.

2. World Health Organization. Global Health Estimates 2015: Disease burden by Cause, Age, Sex, by Country and by Region, 2000-2015. Geneva, 2016.

3. Taylor WD. Clinical practice. Depression in the elderly. *The New England journal of medicine* 2014;371(13):1228-36. doi: 10.1056/NEJMcp1402180 [published Online First: 2014/09/25]

4. Blazer DG. Depression in late life: review and commentary. *The journals of gerontology Series A, Biological sciences and medical sciences* 2003;58(3):249-65. [published Online First: 2003/03/14]

5. Volkert J, Schulz H, Härter M, et al. The prevalence of mental disorders in older people in Western countries – a meta-analysis. *Ageing Research Reviews* 2013;12(1):339-53. doi: http://dx.doi.org/10.1016/j.arr.2012.09.004

6. Lyness JM, Caine ED, King DA, et al. Psychiatric Disorders in Older Primary Care Patients. *Journal of general internal medicine* 1999;14(4):249-54. doi: 10.1046/j.1525-1497.1999.00326.x

7. Jackson JC, Pandharipande PP, Girard TD, et al. Depression, Posttraumatic Stress Disorder, and Functional Disability in Survivors of Critical Illness: results from the BRAIN ICU (Bringing to light the Risk Factors And Incidence of Neuropsychological dysfunction in ICU survivors) Investigation: A Longitudinal Cohort Study. *The lancet Respiratory medicine* 2014;2(5):369-79. doi: 10.1016/S2213-2600(14)70051-7

For peer review only

For peer review only - http://bmjopen.bmj.com/site/about/guidelines.xhtml

8. Gan Y, Gong Y, Tong X, et al. Depression and the risk of coronary heart disease: a meta-analysis of prospective cohort studies. *BMC psychiatry* 2014;14:371. doi: 10.1186/s12888-014-0371-z [published Online First: 2014/12/30]

9. Pan A, Sun Q, Okereke OI, et al. Depression and risk of stroke morbidity and mortality: a meta-analysis and systematic review. *Jama* 2011;306(11):1241-9. doi: 10.1001/jama.2011.1282 [published Online First: 2011/09/22]

10. Cuijpers P, Vogelzangs N, Twisk J, et al. Comprehensive meta-analysis of excess mortality in depression in the general community versus patients with specific illnesses. *The American journal of psychiatry* 2014;171(4):453-62. doi: 10.1176/appi.ajp.2013.13030325 [published Online First: 2014/01/18]

11. Tham A, Jonsson U, Andersson G, et al. Efficacy and tolerability of antidepressants in people aged 65 years or older with major depressive disorder – A systematic review and a meta-analysis. *Journal of affective disorders* 2016;205:1-12. doi: http://dx.doi.org/10.1016/j.jad.2016.06.013

12. Nelson JC, Delucchi K, Schneider LS. Efficacy of second generation antidepressants in late-life depression: a meta-analysis of the evidence. *The American journal of geriatric psychiatry : official journal of the American Association for Geriatric Psychiatry* 2008;16(7):558-67. doi: 10.1097/JGP.0b013e3181693288 [published Online First: 2008/07/02]

13. Thorlund K, Druyts E, Wu P, et al. Comparative efficacy and safety of selective serotonin reuptake inhibitors and serotonin-norepinephrine reuptake inhibitors in older adults: a network meta-analysis. *Journal of the American Geriatrics Society* 2015;63(5):1002-9. doi: 10.1111/jgs.13395 [published Online First: 2015/05/07]

14. Tedeschini E, Levkovitz Y, Iovieno N, et al. Efficacy of antidepressants for late-life depression: a meta-analysis and meta-regression of placebo-controlled randomized

For peer review only

For peer review only - http://bmjopen.bmj.com/site/about/guidelines.xhtml

trials. *The Journal of clinical psychiatry* 2011;72(12):1660-8. doi: 10.4088/JCP.10r06531 [published Online First: 2012/01/17]

15. Gould RL, Coulson MC, Howard RJ. Cognitive behavioral therapy for depression in older people: a meta-analysis and meta-regression of randomized controlled trials. *Journal of the American Geriatrics Society* 2012;60(10):1817-30. doi: 10.1111/j.1532-5415.2012.04166.x [published Online First: 2012/09/26]
16. Kirkham JG, Choi N, Seitz DP. Meta-analysis of problem solving therapy for the treatment of major depressive disorder in older adults. *International journal of geriatric psychiatry* 2016;31(5):526-35. doi: 10.1002/gps.4358 [published Online First: 2015/10/06]
17. Cuijpers P, van Straten A, Smit F. Psychological treatment of late-life depression: a meta-analysis of randomized controlled trials. *International journal of geriatric psychiatry* 2006;21(12):1139-49. doi: 10.1002/gps.1620 [published Online First: 2006/09/07]
18. Cuijpers P, Karyotaki E, Pot AM, et al. Managing depression in older age: psychological interventions. *Maturitas* 2014;79(2):160-69. doi: 10.1016/j.maturitas.2014.05.027
19. Linde K, Sigterman K, Kriston L, et al. Effectiveness of Psychological Treatments for Depressive Disorders in Primary Care: Systematic Review and Meta-Analysis. *The Annals of Family Medicine* 2015;13(1):56-68. doi: 10.1370/afm.1719
20. Holvast F, Massoudi B, Oude Voshaar RC, et al. Non-pharmacological treatment for depressed older patients in primary care: A systematic review and meta-analysis. *PloS one* 2017;12(9):e0184666. doi: 10.1371/journal.pone.0184666
21. Apostolo J, Bobrowicz-Campos E, Rodrigues M, et al. The effectiveness of non-pharmacological interventions in older adults with depressive disorders: A systematic review. *International journal of nursing studies* 2016;58:59-70. doi: 10.1016/j.ijnurstu.2016.02.006 [published Online First: 2016/04/19]

For peer review only - http://bmjopen.bmj.com/site/about/guidelines.xhtml

22. Sukhato K, Lotrakul M, Dellow A, et al. Efficacy of home-based non-pharmacological interventions for treating depression: a systematic review and network meta-analysis of randomised controlled trials. *BMJ open* 2017;7(7)

23. Tonin FS, Rotta I, Mendes AM, et al. Network meta-analysis: a technique to gather evidence from direct and indirect comparisons. *Pharmacy practice* 2017;15(1):943. doi: 10.18549/PharmPract.2017.01.943 [published Online First: 2017/05/16]

24. Moher D, Shamseer L, Clarke M, et al. Preferred reporting items for systematic review and meta-analysis protocols (PRISMA-P) 2015 statement. *Systematic reviews* 2015;4:1. doi: 10.1186/2046-4053-4-1 [published Online First: 2015/01/03]

25. Shamseer L, Moher D, Clarke M, et al. Preferred reporting items for systematic review and meta-analysis protocols (PRISMA-P) 2015: elaboration and explanation. *BMJ (Clinical research ed)* 2015;349:g7647. doi: 10.1136/bmj.g7647 [published Online First: 2015/01/04]

26. MacQueen GM, Frey BN, Ismail Z, et al. Canadian Network for Mood and Anxiety Treatments (CANMAT) 2016 Clinical Guidelines for the Management of Adults with Major Depressive Disorder: Section 6. Special Populations: Youth, Women, and the Elderly. *Canadian journal of psychiatry Revue canadienne de psychiatrie* 2016;61(9):588-603. doi: 10.1177/0706743716659276 [published Online First: 2016/08/04]

27. Kennedy SH, Lam RW, McIntyre RS, et al. Canadian Network for Mood and Anxiety Treatments (CANMAT) 2016 Clinical Guidelines for the Management of Adults with Major Depressive Disorder: Section 3. Pharmacological Treatments. *Canadian journal of psychiatry Revue canadienne de psychiatrie* 2016;61(9):540-60. doi: 10.1177/0706743716659417 [published Online First: 2016/08/04]

For peer review only - http://bmjopen.bmj.com/site/about/guidelines.xhtml

28. Parikh SV, Quilty LC, Ravitz P, et al. Canadian Network for Mood and Anxiety Treatments (CANMAT) 2016 Clinical Guidelines for the Management of Adults with Major Depressive Disorder: Section 2. Psychological Treatments. *Canadian journal of psychiatry Revue canadienne de psychiatrie* 2016;61(9):524-39. doi: 10.1177/0706743716659418 [published Online First: 2016/08/04]

29. Milev RV, Giacobbe P, Kennedy SH, et al. Canadian Network for Mood and Anxiety Treatments (CANMAT) 2016 Clinical Guidelines for the Management of Adults with Major Depressive Disorder: Section 4. Neurostimulation Treatments. *Canadian journal of psychiatry Revue canadienne de psychiatrie* 2016;61(9):561-75. doi: 10.1177/0706743716660033 [published Online First: 2016/08/04]

30. Ravindran AV, Balneaves LG, Faulkner G, et al. Canadian Network for Mood and Anxiety Treatments (CANMAT) 2016 Clinical Guidelines for the Management of Adults with Major Depressive Disorder: Section 5. Complementary and Alternative Medicine Treatments. *Canadian journal of psychiatry Revue canadienne de psychiatrie* 2016;61(9):576-87. doi: 10.1177/0706743716660290 [published Online First: 2016/08/04]

31. Higgins JPT, Sterne JAC, Savović J, et al. A revised tool for assessing risk of bias in randomized trials. In: Chandler J, McKenzie J, Boutron I, et al., eds. Cochrane Methods Cochrane Database of Systematic Reviews 2016, Issue 10 (Suppl 1).

32. DerSimonian R, Laird N. Meta-analysis in clinical trials. *Controlled clinical trials* 1986;7(3):177-88. [published Online First: 1986/09/01]

33. Higgins JP, Thompson SG, Spiegelhalter DJ. A re-evaluation of random-effects meta-analysis. *Journal of the Royal Statistical Society Series A, (Statistics in Society)* 2009;172(1):137-59. doi: 10.1111/j.1467-985X.2008.00552.x [published Online First: 2009/04/22]

For peer review only - http://bmjopen.bmj.com/site/about/guidelines.xhtml

34. Guyatt G. Users' Guides to the Medical Literature: A Manual for Evidence-Based Clinical Practice, 3E: McGraw-Hill Education 2014.

35. Mantel N, Haenszel W. Statistical aspects of the analysis of data from retrospective studies of disease. *Journal of the National Cancer Institute* 1959;22(4):719-48. [published Online First: 1959/04/01]

36. Higgins JP, Thompson SG, Deeks JJ, et al. Measuring inconsistency in meta-analyses. *BMJ (Clinical research ed)* 2003;327(7414):557-60. doi: 10.1136/bmj.327.7414.557 [published Online First: 2003/09/06]

37. Burnham KP, Anderson DR. Model Selection and Multimodel Inference: A Practical Information-Theoretic Approach: Springer New York 2003.

38. Dias S, Welton NJ, Caldwell DM, et al. Checking consistency in mixed treatment comparison meta-analysis. *Statistics in medicine* 2010;29(7-8):932-44. doi: 10.1002/sim.3767 [published Online First: 2010/03/10]

39. van Valkenhoef G, Dias S, Ades AE, et al. Automated generation of node-splitting models for assessment of inconsistency in network meta-analysis. *Research synthesis methods* 2016;7(1):80-93. doi: 10.1002/jrsm.1167 [published Online First: 2015/10/16]

40. Chaimani A, Higgins JP, Mavridis D, et al. Graphical tools for network meta-analysis in STATA. *PloS one* 2013;8(10):e76654. doi: 10.1371/journal.pone.0076654 [published Online First: 2013/10/08]

41. Peters JL, Sutton AJ, Jones DR, et al. Assessing publication bias in meta-analyses in the presence of between-study heterogeneity. *Journal of the Royal Statistical Society: Series A (Statistics in Society)* 2010;173(3):575-91. doi: 10.1111/j.1467-985X.2009.00629.x

For peer review only - http://bmjopen.bmj.com/site/about/guidelines.xhtml

42. Chaimani A, Salanti G. Visualizing assumptions and results in network meta-analysis: The network graphs package. *Stata Journal* 2015;15(4):905-50.

43. Schunemann H, Brozek J, Guyatt G, et al. GRADE handbook for grading quality of evidence and strength of recommendation 2013. Available from: http://gdt.guidelinedevelopment.org/app/ (assessed 22 Nov 2016).

44. Hutton B, Salanti G, Caldwell DM, et al. The PRISMA extension statement for reporting of systematic reviews incorporating network meta-analyses of health care interventions: checklist and explanations. *Annals of internal medicine* 2015;162(11):777-84. doi: 10.7326/m14-2385 [published Online First: 2015/06/02]

For peer review only

For peer review only - http://bmjopen.bmj.com/site/about/guidelines.xhtml

PRISMA-P (Preferred Reporting Items for Systematic review and Meta-Analysis Protocols) 2015 checklist: recommended items to address in a systematic review protocol*

Section and topic	Item No	Checklist item	Page (Line)
**ADMINISTRATIVE INFORMATION**			
Title:			
Identification	1a	Identify the report as a protocol of a systematic review	1 (2)
Update	1b	If the protocol is for an update of a previous systematic review, identify as such	Not applicable
Registration	2	If registered, provide the name of the registry (such as PROSPERO) and registration number	3 (5)
Authors:			
Contact	3a	Provide name, institutional affiliation, e-mail address of all protocol authors; provide physical mailing address of corresponding author	1 (11)
Contributions	3b	Describe contributions of protocol authors and identify the guarantor of the review	Not applicable
Amendments	4	If the protocol represents an amendment of a previously completed or published protocol, identify as such and list changes; otherwise, state plan for documenting important protocol amendments	Not applicable
Support:			
Sources	5a	Indicate sources of financial or other support for the review	17 (10)
Sponsor	5b	Provide name for the review funder and/or sponsor	Not applicable
Role of sponsor or funder	5c	Describe roles of funder(s), sponsor(s), and/or institution(s), if any, in developing the protocol	17 (12)
**INTRODUCTION**			
Rationale	6	Describe the rationale for the review in the context of what is already known	5 (5)
Objectives	7	Provide an explicit statement of the question(s) the review will address with reference to participants, interventions, comparators, and outcomes (PICO)	5 (24)
**METHODS**			
Eligibility criteria	8	Specify the study characteristics (such as PICO, study design, setting, time frame) and report characteristics (such as years considered, language, publication status) to be used as criteria for eligibility for the review	6 (14)
Information sources	9	Describe all intended information sources (such as electronic databases, contact with study authors, trial registers or other grey literature sources) with planned dates of coverage	9 (8)
Search strategy	10	Present draft of search strategy to be used for at least one electronic database, including planned limits, such that it could be repeated	9 (11)

For peer review only

Study records:			
Data management	11a	Describe the mechanism(s) that will be used to manage records and data throughout the review	10 (4)
Selection process	11b	State the process that will be used for selecting studies (such as two independent reviewers) through each phase of the review (that is, screening, eligibility and inclusion in meta-analysis)	10 (5)
Data collection process	11c	Describe planned method of extracting data from reports (such as piloting forms, done independently, in duplicate), any processes for obtaining and confirming data from investigators	11 (14)
Data items	12	List and define all variables for which data will be sought (such as PICO items, funding sources), any pre-planned data assumptions and simplifications	11 (16)
Outcomes and prioritization	13	List and define all outcomes for which data will be sought, including prioritization of main and additional outcomes, with rationale	12 (16)
Risk of bias in individual studies	14	Describe anticipated methods for assessing risk of bias of individual studies, including whether this will be done at the outcome or study level, or both; state how this information will be used in data synthesis	12 (6)
Data synthesis	15a	Describe criteria under which study data will be quantitatively synthesised	Not applicable
	15b	If data are appropriate for quantitative synthesis, describe planned summary measures, methods of handling data and methods of combining data from studies, including any planned exploration of consistency (such as I^2, Kendall's τ)	13 (7)
	15c	Describe any proposed additional analyses (such as sensitivity or subgroup analyses, meta-regression)	13 (14)
	15d	If quantitative synthesis is not appropriate, describe the type of summary planned	Not applicable
Meta-bias(es)	16	Specify any planned assessment of meta-bias(es) (such as publication bias across studies, selective reporting within studies)	14 (22)
Confidence in cumulative evidence	17	Describe how the strength of the body of evidence will be assessed (such as GRADE)	15 16)

*** It is strongly recommended that this checklist be read in conjunction with the PRISMA-P Explanation and Elaboration (cite when available) for important clarification on the items. Amendments to a review protocol should be tracked and dated. The copyright for PRISMA-P (including checklist) is held by the PRISMA-P Group and is distributed under a Creative Commons Attribution Licence 4.0.**

From: Shamseer L, Moher D, Clarke M, Ghersi D, Liberati A, Petticrew M, Shekelle P, Stewart L, PRISMA-P Group. Preferred reporting items for systematic review and meta-analysis protocols (PRISMA-P) 2015: elaboration and explanation. BMJ. 2015 Jan 2;349(jan02 1):g7647.

For peer review only
